# The edible seaweed *Laminaria japonica* contains cholesterol analogues that inhibit lipid peroxidation and cyclooxygenase enzymes

**DOI:** 10.1371/journal.pone.0258980

**Published:** 2022-01-27

**Authors:** Xingyu Lu, Amila A. Dissanayake, Chuqiao Xiao, Jie Gao, Mouming Zhao, Muraleedharan G. Nair

**Affiliations:** 1 School of Light Industry and Food Engineering, Guangxi University, Nanning, China; 2 Department of Horticulture, Bioactive Natural Products and Phytoceuticals Laboratory, Michigan State University, East Lansing, Michigan, United States of America; 3 School of Food Science and Engineering, South China University of Technology, Guangzhou, China; 4 Chaozhou Branch of Chemistry and Chemical Engineering Guangdong Laboratory, Chaozhou, China; Tianjin University of Traditional Chinese Medicine, CHINA

## Abstract

In this study, 5 sterols were isolated and purified from *Laminaria japonica*, commonly known as edible brown seaweed, and their structures were identified based on detailed chemical methods and spectroscopic analyses. Spectroscopic analyses characterized 5 sterols as 29-Hydroperoxy-stigmasta-5,24(28)-dien-3β-ol, saringosterol (24-vinyl-cholest-5-ene-3β,24-diol), 24-methylenecholesterol, fucosterol (stigmasta-5,24-diene-3β-ol), and 24-Hydroperoxy-24-vinyl-cholesterol. The bioactivities of these sterols were tested using lipid peroxidation (LPO) and cyclooxygenase (COX-1 and -2) enzyme inhibitory assays. Fucosterol exhibited the highest COX-1 and -2 enzyme inhibitory activities at 59 and 47%, respectively. Saringosterol, 24-methylenecholesterol and fucosterol showed higher LPO inhibitory activity at >50% than the other compounds. In addition, the results of molecular docking revealed that the 5 sterols were located in different pocket of COX-1 and -2 and fucosterol with tetracyclic skeletons and olefin methine achieved the highest binding energy (-7.85 and -9.02 kcal/mol) through hydrophobic interactions and hydrogen bond. Our results confirm the presence of 5 sterols in *L*. *japonica* and its significant anti-inflammatory and antioxidant activity.

## 1. Introduction

*Laminaria japonica*, a commercial edible brown seaweed, is widely cultivated and consumed as a health food resource in China, Japan, and Korea. Over the past decades, *L*. *japonica* is one of the most important marine medicinal foodstuffs and a rich source of various functional compounds, which have been widely investigated by both *in vitro* and *in vivo* experiments [1–4]. Numerous phytochemical investigations on *L*. *japonica* revealed the presence of polyphenols, fatty acids, carotenoids, steroidal ketones, amine, sterols and tocopherol [5–13]. Fucoxanthin, one of the most abundant carotenoids and the major functional pigment present in *L*. *japonica*, has been reported to have antioxidative, cytoprotective, anti-inflammatory and anti-obese properties [6–9]. Butyl-isobutyl-phthalate, extracted from *L*. *japonica*, exhibited hypoglycemic effect in vivo and non-competitive inhibition of α-glucosidase in vitro [10, 11]. Six isolates from EtOAc extracts of *L*. *japonica* have been determined as 2,6-dibromo-4-(2-(methylamino)ethyl)phenol, 6-bromo-1H-indole-3-carbaldehyde, 1H-indole-3-carbaldehyde, 4-bromobenzoic aldehyde, 4-bromobenzoic acid and 4-hydroxybenzoic acid [12]. Four steroidal ketones including ergosta-4,24(28)-diene-3-one, ergosta-4,24(28)-diene-3,6-dione, stigmasta-4,24(28)-diene-3-one and stigmasta-4,24(28)-diene-3,6-dione have also been isolated from *L*. *japonica* [13].

Oxidative stress can activate a variety of transcription factors, which lead to the differential expression of some genes involved in inflammatory pathways [14]. The oxidative stress machinery and inflammatory signaling are not only interrelated, but their impairment can lead to many chronic diseases. Previous phytochemical investigations revealed the hexane extract from *L*. *japonica* exerts anti-inflammatory effects [15]. Nevertheless, limited studies on the purification of hexane extracts from *L*. *japonica* and structure characterization of the pure compounds have been performed and, to the best of our knowledge, anti-inflammatory and antioxidant activities of these pure compounds have not been reported. Herein, 5 sterols from hexane extracts of *L*. *japonica* have been purified by VLC (vacuum liquid chromatography), multi-step MPLC (medium-pressure liquid chromatography), Prep-TLC (preparative thin-layer chromatography) and HPLC (high pressure liquid chromatography), and characterized by high-resolution electrospray ionization mass spectrometry (HRESITOFMS), nuclear magnetic resonance (NMR) and gas chromatography-mass spectrometry (GC-MS) analyses. Lipid peroxidation and cyclooxygenase enzyme inhibitory of these sterols were determined by using lipid peroxidation (LPO) and cyclooxygenase enzymes (COX-1 and -2) inhibitory assays according to our previous study [16–21]. Moreover, molecular docking analysis was performed to investigate the structure-activity relationships of these sterols.

## 2. Materials and methods

### 2.1 General experimental procedures

Waters 2010 HPLC (high pressure liquid chromatography) system (Waters Corp., Milford, MA, USA) equipped with XTerra Prep MS C-18 column (10 μm, 19 x 250 mm) (Waters Corporation, Milford, MA, USA) was used for the HPLC purification and all solvents used for HPLC purification were of ACS reagent grade (Sigma-Aldrich Chemical Company, St. Louis, MO, USA). The NMR (Nuclear magnetic resonance) spectra were obtained using Agilent DirectDrive2 500 MHz (Agilent Technologies, Palo Alto, CA, USA) in deuterated chloroform (CDCl_3_) purchased from Cambridge Isotope Laboratories, Inc. The ^1^H and ^13^C chemical shift values were expressed in ppm, based on the residual chemical shift values, for CDCl_3_ at *δ*H 7.24 and *δ*C 77.2 ppm, respectively. The COX-1 enzyme was prepared from ram seminal vesicles purchased from Oxford Biomedical Research, Inc. (Oxford, MI). The COX-2 enzyme was prepared from insect cells cloned with human PGHS-2 enzyme. Positive controls for the COX and LPO assays aspirin, naproxen, Celebrex^®^, ibuprofen, TBHQ (t-butylhydroquinone), BHA (butylated hydroxyanisole), BHT (butylated hydroxytoluene) and cholesterol were purchased from Sigma-Aldrich Chemical Company (St. Louis, MO, USA) and arachidonic acid was purchased from Oxford Biomedical Research, Inc (Oxford Biomedical Research, Inc., Oxford, MI, USA). The fluorescent probe, 3–[*p*-(6-phenyl)-1,3,5-hexatrienyl] phenylpropionic acid was purchased from Molecular Probes (Eugene, OR, USA) and 1-stearoyl-2-linoleoyl-sn-glycerol-3-phosphocholine (SLPC) was purchased from Avanti Polar Lipids (Alabaster, AL, USA).

### 2.2 Plant materials

*L*. *japonica* (GS-02-004-2013) used in this study were cultivated at Yantai (Shandong province, China) and harvested in July 2018. Fresh raw *L*. *japonica* was dried immediately after harvest under sun light and then shipped to the lab. The raw *L*. *japonica* in the lab was first washed with running tap water and deionized water, and then dried in an oven at 50°C. The dry sample was further pulverized with a blender to obtain the dry *L*. *japonica* power through a 50-mesh screen.

### 2.3 Extraction and isolation

The dry *L*. *japonica* power (5 kg) was extracted with Hexane (10 L, 12 h×3) and evaporated under vacuum to afford hexanes extract HE (22 g). An aliquot of HE (13 g) was pretreated with activated charcoal and then fractionated using silica gel VLC by eluting with hexanes-ethyl acetate (4:1, 2:1 and 1:1, v/v), followed by methanol (100%), to yield fractions A (855 mg), B (4 g), C (1.44 g), D (374 mg), E (205 mg), and F (3 g), respectively. An aliquot of fractions A (68 mg) was fractionated by Pre-TLC with hexanes-acetone (10: 1, *v/v*) to yield the main fraction A-1(31 mg). An aliquot of fractions B (76 mg) was fractionated by Pre-TLC with hexanes-acetone (8: 1, *v/v*) to yield the main fraction B-1(50 mg), which was characterized as fatty acid and hence were kept aside. Fraction D (374 mg) fractionated by MPLC with hexanes-acetone (2: 1, and 1: 1, *v/v*) and Pre-TLC with chloroform: methanol (40: 1, *v/v*) yielded three main fractions D-1(24 mg, 86B), D-2(2 mg, 91C) and D-3(20 mg, 72B), which were the same compounds that from fraction C. And fraction E and F were found to be salt and put away.

Fraction C (1.44 g) was further purified with silica gel MPLC by eluting with hexanes-acetone (4: 1, 2:1 and 1: 1, v/v), to yield fractions G (301 mg), H (599 mg), I (137 mg), J (160 mg), and K (166 mg), respectively. Fraction G had been put away for it was too complicated.

An aliquot of the main fraction **H** (215 mg) was fractionated by silica gel VLC and eluted under gradient conditions using chloroform: methanol (100:1 and 4.1 *v/v*) to yield fractions **LJ-1** (161 mg) and **LJ-2** (51 mg). An aliquot of fraction **LJ-1** (160 mg), purified by HPLC by eluting with acetonitrile: methanol 50:50 *v/v*, 4 mL/min under isocratic conditions yielded fraction **LJ-1(a)** (62 mg), compound **3** (39 mg, 42 min, 24-methylenecholesterol, S1 Fig) and compound **4** (58 mg, 51 min, fucosterol, S1 Fig) [22–24]. Repeated HPLC purification of the fraction **LJ-1(a)** (40 mg) eluting with acetonitrile:methanol 95:5 *v/v*, 3 mL/min under isocratic conditions yielded compound **1** (12 mg, 46 min, 29-Hydroperoxy-stigmasta-5,24(28)-dien-3β-ol, S1 Fig) and compound **2** (22 mg, 51 min, saringosterol S1 Fig) [22].

Fraction I (137 mg) was purified by Prep-TLC on silica gel with chloroform-Methanol (40: 1) as the mobile phase, yielded fraction L (33 mg). An aliquot of fraction **L** (20 mg) was further separated by recrystallization from hexane: acetone to afford compound **5** (17 mg, 24-hydroperoxy-24-vinyl-cholesterol) [22].

Pre-TLC with chloroform: methanol (60: 1, *v/v*) of fraction J (51 mg) produced J-1 (15 mg, 87B) and J-2 (8 mg, 87D). Fraction K (75 mg) fractionated by Pre-TLC with hexanes-acetone (8: 1, *v/v*) afforded K-1 (43 mg, 69B). Fraction A-1, B-1, J-1, J-2, and K-1 mainly contained fatty acids and triglycerides based on preliminary analyses were kept aside due to prior published work [25].

### 2.4 Cyclooxygenase enzymes (COX-1 and -2) inhibitory assays

The COX-1 and-2 enzyme inhibitory activity of pure isolates (compounds 1–5) from *L*. *japonica* and cholesterol were measured by monitoring the initial rate of O_2_ uptake using an Instech micro oxygen chamber and electrode (Instech Laboratories) attached to a YSI model 5300 biological oxygen monitor (Yellow Springs Instrument, Inc.) at 37°C according to the published procedure. Commercial NSAIDs aspirin, ibuprofen, Celebrex^®^ and naproxen were used as positive control groups to verify the sensitivity of the experiment [25–28].

### 2.5 Lipid peroxidation inhibitory assay

The antioxidant activity of all isolates (compounds **1**–**5**) and cholesterol was determined by the LPO inhibitory assay using fluorescence spectroscopy on a Turner model 450 fluorometer (Barnstead/Thermolyne Corp.) according to the reported procedure. Commercial antioxidants BHA, BHT and TBHQ were used as positive control groups to verify the sensitivity of the experiment [17–20].

### 2.6 Molecular docking

Docking was performed to investigate the molecular interactions between the pure compounds and cyclooxygenase using the AutoDock Vina open-source program (ver. 1.1.2) according to the reported procedure [29, 30]. The crystallographic structures of ovine COX-1 (PDB: 3N8Z) and human COX-2 (PDB: 5F1A) were retrieved from the Protein Data Bank in SDF format. The 3D conformations of compounds 2–4 (CID: 14161394, 92113, 5281328) were provided from PubChem (http://pubchem.ncbi.nlm.nih.gov/). The 3D conformations of compounds 1 and 5 were drawn at MolView (https://molview.org/). Virtual screening of all compounds was performed into the active site of the COX-1 and COX-2 domain by using AutoDock Vina. The whole protein structures were targeted for compounds docking [31]. Molecular docking coordinates for COX-1 receptor were determined as center_x = -34.054, center_y = 56.874, center_z = -11.092, dimensions parameters are size_x = 94, size_y = 96, size_z = 126, spacing parameter is 0.808 Å. Docking parameters used for COX-2 receptor were center_x = 34.099, center_y = 27.4, center_z = 217.829, size_x = 96, size_y = 96, size_z = 126, spacing parameter is 0.789 Å. Cluster analysis was performed on the docked results using an RMS tolerance of 2.0 Å. The docked formation with the lowest energy value was obtained, and enzyme-ligand interactions were visualized using open-source PyMOL created by Warren Lyford DeLano (https://github.com/schrodinger/pymol-open-source).

### 2.7 Statistical analysis

The data was presented as mean ± SD (n = 3) and evaluated by one-way analysis of variance (ANOVA) followed by the Duncan’s test. All statistical analyses were carried out using R statistical package and SPSS for Windows, Version 17.0 (SPSS Inc., Chicago, IL, USA).

## 3. Results and discussion

### 3.1 Structure elucidation

**Fig 1:**
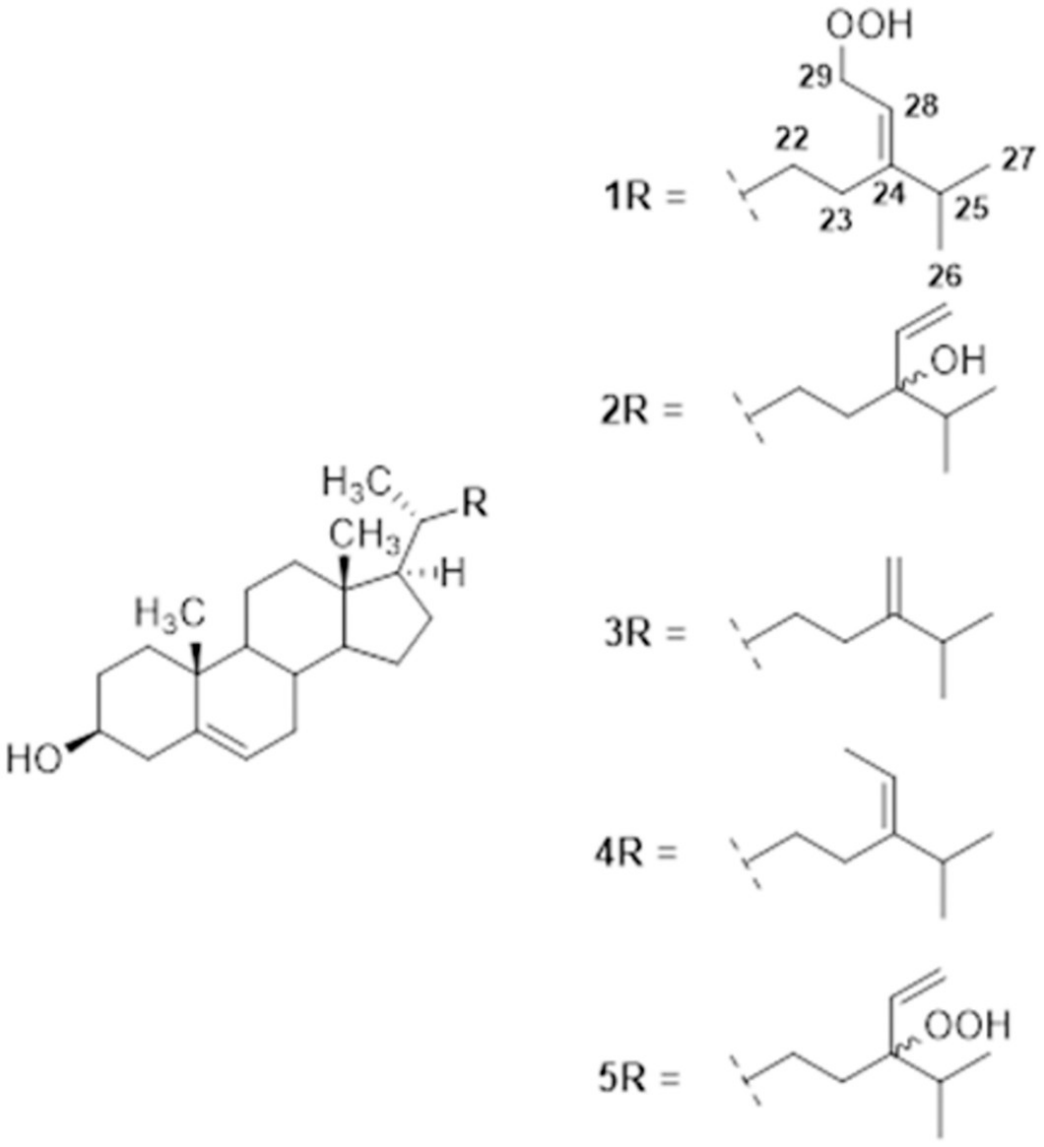
Chemical structures of the biologically active sterols (compounds 1–5) isolated from the hexane extract of *L*. *japonica*.

Compound **1** was isolated as a white powder and exhibited a molecular ion peak at *m/z* 427.3563 [M-H_2_O+H]^+^ in its positive-ion HRESITOFMS consistent with the molecular formula C_29_H_48_O_3_, This confirmed six indices of hydrogen deficiency in compound **1** (S2 Fig). The ^1^H-NMR (500 MHz, CDCl_3_) spectrum exhibited two olefin methine proton signal at *δ*H 5.33 (1H, brd, J = 4.9 Hz, H-6) and *δ*H 4.53 (1H, d, J = 6.8 Hz, H-28), one oxygenated methine proton signal at *δ*H 3.49 (1H, m, H-3), and five methyl proton signals at *δ*H 0.99 (3H, s, H-19), 0.97 (3H, d, J = 6.8 Hz, H-21), 0.95 (3H, d, J = 6.8 Hz, H-27), 0.89 (3H, d, J = 6.7 Hz, H-26), and 0.65 (3H, s, H-18) (S3 Fig). The ^13^C-NMR (125 MHz, CDCl_3_) spectrum exhibited 29 carbon signals including 2 olefin quaternary carbon signals at *δ*C 155.3 (C-24) and 140.7 (C-5), two olefin methine carbon signals at *δ*C121.7 (C-6) and *δ*C114.8 (C-28), one oxygenated methine carbon signal at *δ*C 71.8 (C-3), one oxygenated methylene carbon signal at *δ*C 73.5 (C-29) and 5 methyl carbon signals at *δ*C 21.9 (C-26), 21.8 (C-27), 19.4 (C-19), 18.7 (C-21), and 11.8 (C-18) (S4 Fig). According to the spectral data, compound **1** was identified as 29-Hydroperoxy-stigmasta-5,24(28)-dien-3β-ol (Fig 1) [22].

Compound **2** was isolated as a white powder and exhibited a molecular ion peak at *m/z* 427.3562 [M+H-2H]^+^ in its positive-ion HRESITOFMS consistent with the molecular formula C_29_H_48_O_2_, This confirmed six indices of hydrogen deficiency in compound **2** (S5 Fig). The ^1^H-NMR (500 MHz, CDCl_3_) spectrum exhibited two olefin methine proton signals at δH 5.34 (1H, brd, J = 4.9 Hz, H-6) and 5.75 (1H, m, H-28), one olefin methylene signal at δH 5.11–5.27 (2H, m, H-29a and H-29b), one oxygenated methine proton signal at δH 3.49 (1H, m, H-3), and five methyl proton signals at δH 0.98 (3H, s, H-19), 0.90 (3H, d, J = 6.8 Hz, H-21), 0.89 (3H, d, J = 6.8 Hz, H-27), 0.85 (3H, d, J = 6.7 Hz, H-26), and 0.66 (3H, s, H-18) (S6 Fig). The ^13^C-NMR (125 MHz, CDCl_3_) spectrum exhibited 29 carbon signals including one olefin quaternary carbon signal at δC 140.7 (C-5), one olefin methylene carbon signals at *δ*C121.7 (C-29), two olefinic methines carbon signals at *δ*C137.1 (C-5) and *δ*C116.3 (C-28), two oxygenated methine carbon signal at δC 71.7 (C-3) and δC 89.1 (C-24), and five methyl carbon signals at δC 21.0 (C-26), 19.3 (C-27), 18.7 (C-19), 17.5 (C-21), and 11.8 (C-18) (S7 Fig). Therefore, according to the spectral data, compound **2** was identified as saringosterol (24-vinyl-cholest-5-ene-3β,24-diol) (Fig 1) [22].

Compound **3** was isolated as a white powder and exhibited a molecular ion peak at *m/z* 381.3507 [M-H_2_O+H]^+^ in its positive-ion HRESITOFMS consistent with the molecular formula C_28_H_46_O, This confirmed six indices of hydrogen deficiency in compound **3** (S8 Fig). The ^1^H-NMR (500 MHz, CDCl_3_) spectrum exhibited one olefin methine proton signal at *δ*H 5.33 (1H, brd, J = 4.9 Hz, H-6), one olefinic methylene proton signals at *δ*H 4.69 (1H, d, J = 1.0, H-28a) and 4.64 (1H, d, J = 1.0, H-28b), one oxygenated methine proton signal at *δ*H 3.49 (1H, m, H-3), and five methyl proton signals at *δ*H 0.98 (3H, s, H-19), 0.97 (3H, d, J = 6.8 Hz, H-21), 0.96 (3H, d, J = 6.8 Hz, H-27), 0.94 (3H, d, J = 6.7 Hz, H-26), and 0.66 (3H, s, H-18) (S9 Fig). The ^13^C-NMR (125 MHz, CDCl_3_) spectrum exhibited 28 carbon signals including 2 olefin quaternary carbon signals at *δ*C 156.9 (C-24) and 140.7 (C-5), one olefin methine carbon signals at *δ*C121.7 (C-6), one olefin methylene carbon signals at *δ*C105.9 (C-28), one oxygenated methine carbon signal at *δ*C 71.8 (C-3), and 5 methyl carbon signals at *δ*C 21.9 (C-26), 21.8 (C-27), 19.4 (C-19), 18.7 (C-21), and 11.8 (C-18) (S10 Fig). According to the spectral data, compound **3** was identified as 24-methylenecholesterol (Fig 1) [22, 23].

Compound **4** was also isolated as a white powder and exhibited a molecular ion peak at *m/z* 395.3669 [M-H_2_O+H]^+^ in its positive-ion HRESITOFMS consistent with the molecular formula C_29_H_48_O, This confirmed six indices of hydrogen deficiency in compound **4** (S11 Fig). The ^1^H-NMR (500 MHz, CDCl_3_) spectrum exhibited two olefin methine proton signals at *δ*H 5.33 (1H, brd, J = 4.9 Hz, H-6) and 5.16 (1H, dd, J = 6.8 and 6.8 Hz, H-28), one oxygenated methine proton signal at *δ*H 3.49 (1H, m, H-3), one allyl methyl proton signal at *δ*H 1.55 (3H, d, J = 6.8 Hz, H-29), and five methyl proton signals at *δ*H 0.98 (3H, s, H-19), 0.97 (3H, d, J = 6.8 Hz, H-21), 0.96 (3H, d, J = 6.8 Hz, H-27), 0.94 (3H, d, J = 6.7 Hz, H-26), and 0.66 (3H, s, H-18) (S12 Fig). The ^13^C-NMR (125 MHz, CDCl_3_) spectrum exhibited 29 carbon signals including 2 olefin quaternary carbon signals at *δ*C 146.9 (C-24) and 140.7 (C-5), two olefin methine carbon signals at *δ*C121.7 (C-6) and 115.5 (C-28), one oxygenated methine carbon signal at *δ*C 71.7 (C-3), and six methyl carbon signals at *δ*C 13.2 (C-29), 22.2 (C-26), 22.1 (C-27), 19.4 (C-19), 18.7 (C-21), and 11.8 (C-18) (S13 Fig). Therefore, according to the spectral data, compound **4** was identified as fucosterol (stigmasta-5,24-diene-3β-ol) (Fig 1) [22, 26]

Compound **5** was also isolated as a white powder and exhibited a molecular ion peak at *m/z* 427.3562 [M-H_2_O+H]^+^ in its positive-ion HRESITOFMS consistent with the molecular formula C_29_H_48_O_3_, This confirmed six indices of hydrogen deficiency in compound **5** (S14 Fig). The ^1^H-NMR (500 MHz, CDCl_3_) spectrum exhibited two olefin methine proton signals at δH 5.34 (1H, brd, J = 4.9 Hz, H-6) and 5.75 (1H, m, H-28), one olefin methylene signal at δH 5.09–5.19 (2H, m, H-29a and H-29b), one oxygenated methine proton signal at δH 3.49 (1H, m, H-3), and five methyl proton signals at δH 0.99 (3H, s, H-19), 0.91 (3H, d, J = 6.8 Hz, H-21), 0.89 (3H, d, J = 6.8 Hz, H-27), 0.86 (3H, d, J = 6.7 Hz, H-26), and 0.64 (3H, s, H-18) (S15 Fig). The ^13^C-NMR (125 MHz, CDCl_3_) spectrum exhibited 29 carbon signals including one olefin quaternary carbon signal at δC 140.7 (C-5), one olefin methylene carbon signals at *δ*C121.6 (C-29), two olefinic methines carbon signals at *δ*C 112.8 (C-5) and *δ*C 142.5 (C-28), two oxygenated methine carbon signal at δC 71.7 (C-3) and δC 77.7 (C-24), and five methyl carbon signals at δC 21.0 (C-26), 19.3 (C-27), 18.7 (C-19), 17.5 (C-21), and 11.8 (C-18) (S16 Fig). Therefore, according to the spectral data, compound **5** was identified as 24-Hydroperoxy-24-vinyl-cholesterol (Fig 1) [22].

### 3.2 Cyclooxygenase enzymes (COX-1 and -2) inhibitory assay

**Fig 2:**
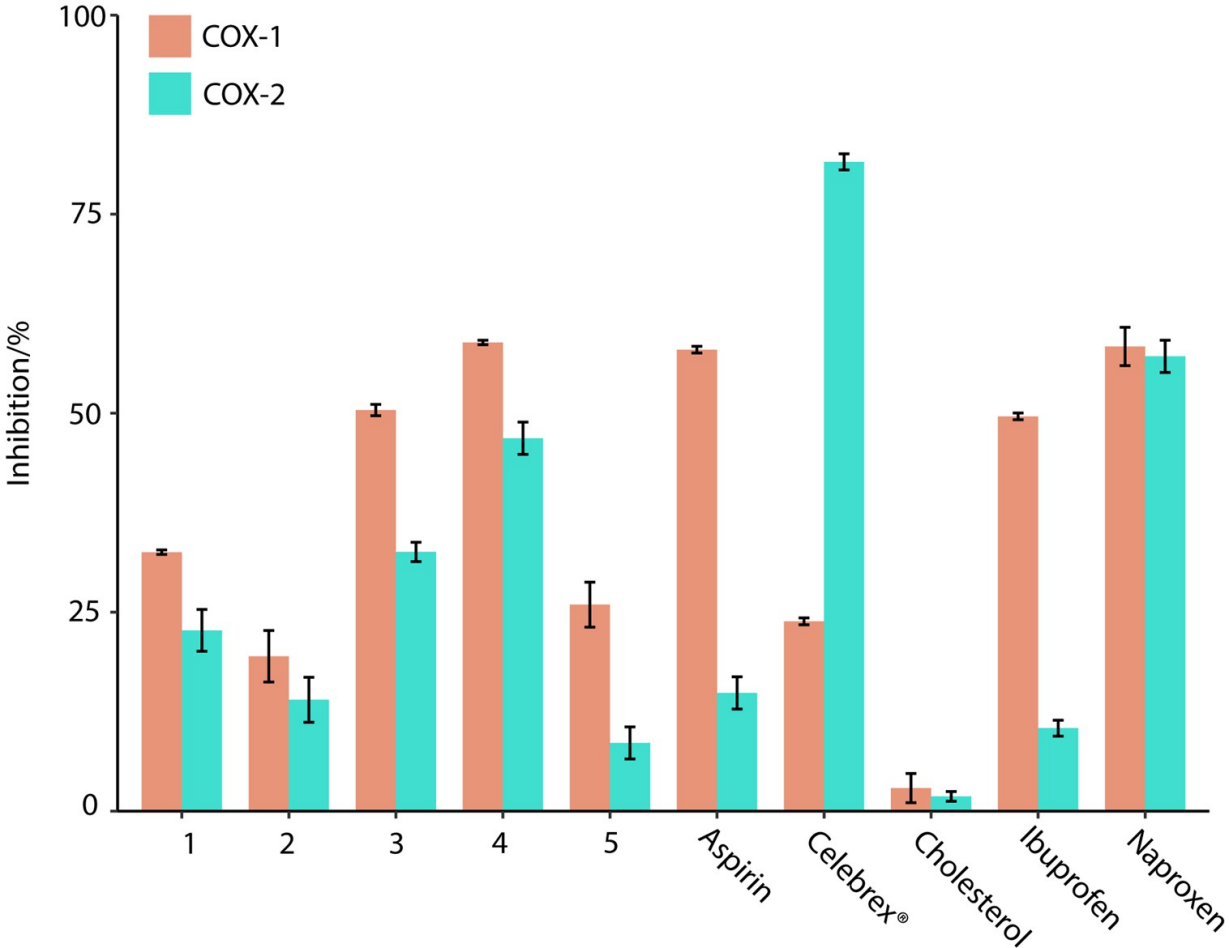
Cyclooxygenase enzymes (COX-1 and -2) enzyme inhibitory activities sterols (1–5) from the hexane extract of *L*. *japonica* and cholesterol were tested at 25 μg/mL concentration. Commercial NSAIDs aspirin, ibuprofen, Celebrex^®^ and naproxen used as positive control and tested at 108, 12, 1 and 12 μg/mL, respectively. The varying concentrations of positive controls used were to yield a comparable activity profile between 50–100% by test compounds and positive controls alike. Vertical bars represent the standard deviation of each data point (n = 2).

Prostaglandins, the inflammation-causing hormones, were converted from arachidonic acid by the catalysis of COX enzymes. Therefore, inhibition of COX enzymes could prevent the production of prostaglandins and reduce inflammation [26, 32]. The anti-inflammatory activity of the pure isolates from *L*. *japonica* was revealed by their COX-1 and -2 enzyme inhibitions. In this study, compounds (**1**–**5**) at 25 μg/mL inhibited COX-1 enzyme by 32, 19, 50, 59, and 26% and COX-2 enzyme by 23, 14, 33, 47, and 9%, respectively (Fig 2). Commercial NSAIDs aspirin, Celebrex^®^, naproxen and ibuprofen were used as positive control at 108, 1, 12, and 15 μg/mL, respectively (Fig 2). Apparently, the COX enzyme inhibitory activity of 24-methylenecholesterol (compound 3) and fucosterol (compound 4) was comparable to the activity of the over-the-counter nonsteroidal anti-inflammatory drugs (NSAIDs) aspirin and ibuprofen. Compound 3 and 4 inhibited the COX-1 enzyme at a higher rate than the COX-2 enzyme, which is similar to aspirin and ibuprofen.

### 3.3 Lipid peroxidation inhibitory assay

**Fig 3:**
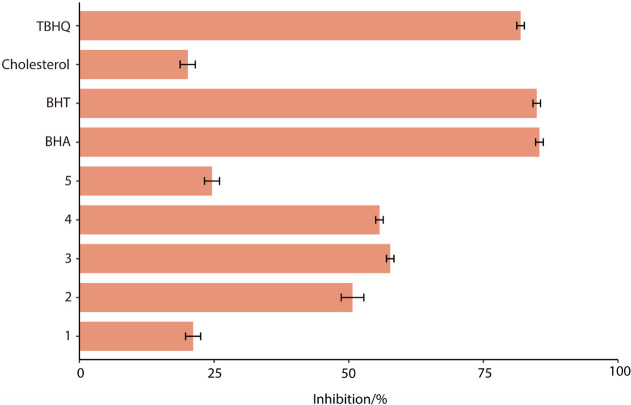
Lipid peroxidation (LPO) inhibitory activities of sterols (1–5) isolated from the hexane extract of *L*. *japonica* and commercial cholesterol tested at 25 μg/mL. Commercial antioxidants BHA, BHT and TBHQ used as positive controls at 1.8, 2.2 and 1.6 μg/mL. The oxidation of lipid was initiated by the addition of Fe^2+^ ions. The varying concentrations of positive controls used were to yield a comparable activity profiles between 50–100% by test compounds and positive controls alike. Vertical bars represent the standard deviation of each data point (n = 2).

Potential antioxidant activity of the pure compounds 1–5 was determined by the LPO assays using the large unilamellar vesicles (LUVs) model system, the results of which are shown in Fig 3. The LPO assay detects compounds that can scavenge free radicals. At 25 μg/mL, the LPO inhibitory activity of sterols **1**–**5** and cholesterol was 21, 51, 58, 56, 25 and 20%, respectively, as compared to the commercial antioxidants BHA, BHT and TBHQ at 85, 85 and 82%, respectively. The concentrations used to test LPO inhibitory activity for compounds were at 1.8, 2.2 and 1.6 μg/mL, respectively (Fig 3). Compared to compound 1 and 5, saringosterol (compound 2), 24-methylenecholesterol (compound 3) and fucosterol (compound 4) showed the higher LPO inhibitory activity at >50%, at 25 μg/mL concentration.

### 3.4 Molecular docking

Molecular docking was used to predict the interactions between the 5 sterols and the COX-1 and -2 enzyme at the molecular level. Root-Mean-Squared-Deviation (RMSD) is a similarity metric between molecular conformations, the lower the RMSD value is, the more similar the molecular conformations of ligands (before and after the docking) is. As shown in Table 1, the RMSD values of all the detected ligands were below the maximum allowed value of 2 Å, indicating ligands underwent minimal deformation during docking and the docking results were credible [33]. Docking results revealed that the binding energy of compounds 1–5 were -7.64, -6.36, -7.79, -7.85 and -6.44 kcal/mol for COX-1, and -8.49, -8.33, -8.72, -9.02 and -6.97 kcal/mol for COX-2, which have the similar trends with their COX-1 and -2 enzyme inhibitory activities (Table 1).

**Table 1:** Docking results of all the 5 sterols against COX-1 and -2 receptors.

Receptor with PDB IDs	Ligand	Binding affinity (kcal/mol)	RMSD(Å)	No. of H-bonds	Amino acid residues forming H-bonds
COX-1 (3N8Z)	Comp. 1	-7.64	1.28	3	ASP-135, ASP-158
	Comp. 2	-6.36	1.43	1	GLU-326
	Comp. 3	-7.79	1.09	1	ASP-158
	Comp. 4	-7.85	1.08	0	None
	Comp. 5	-6.44	1.69	1	LEU-115
COX-2 (5F1A)	Comp. 1	-8.49	0.88	2	GLY-225, TYR-373
	Comp. 2	-8.33	1.82	2	ASN-375
	Comp. 3	-8.72	1.06	1	GLY-225
	Comp. 4	-9.02	1.43	1	GLY-225
	Comp. 5	-6.97	1.83	2	ARG-120, GLU-524

**Fig 4:**
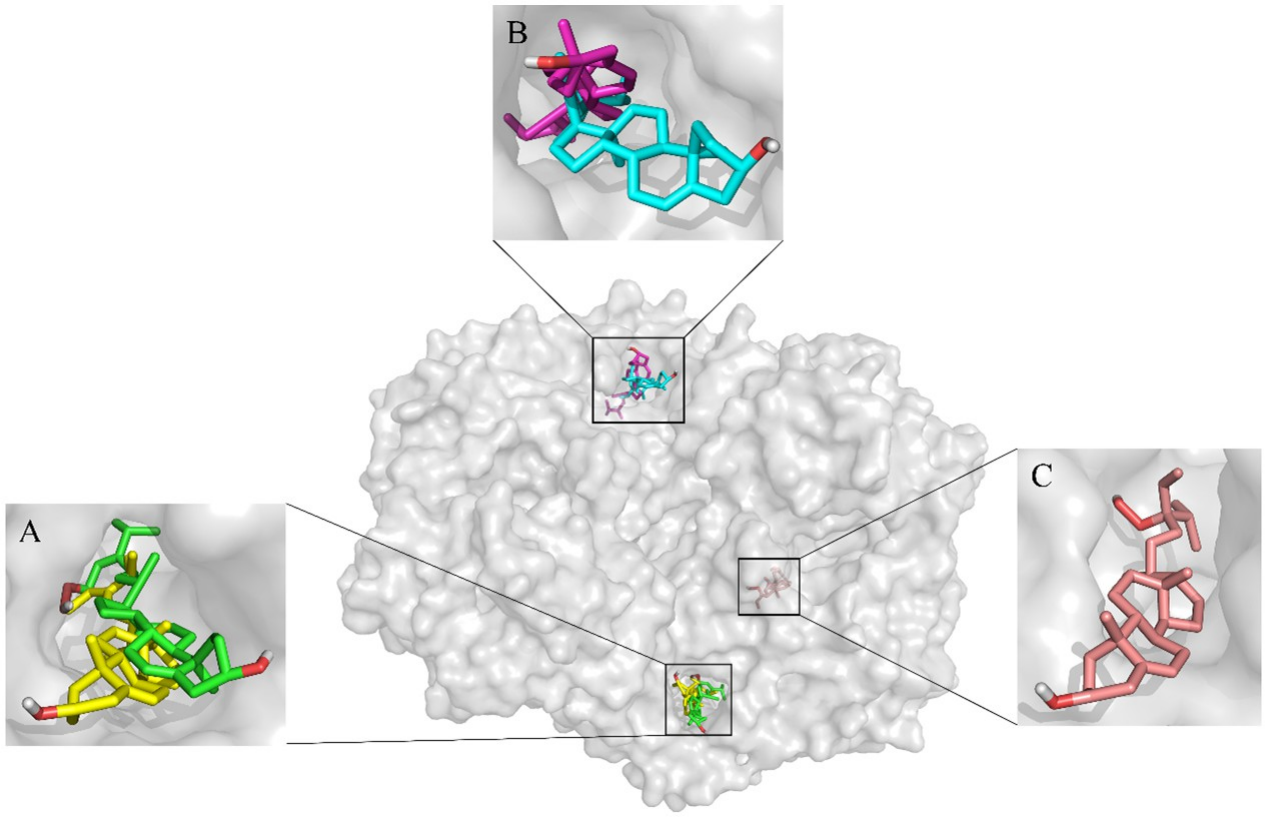
Molecular docking of COX-1 with compounds 1–5. Different compounds were located in different active pockets. Compounds 1 and 4 were located in the hydrophobic pocket A of COX-1 (Comp. 1 shown in green, Comp.4 shown in yellow), compounds 2 and 3 were located in the pocket B of COX-1 (Comp.2 shown in cyan, Comp.3 shown in magenta), while compound 5 was located in the pocket C (Comp.5 shown in salmon).

**Fig 5:**
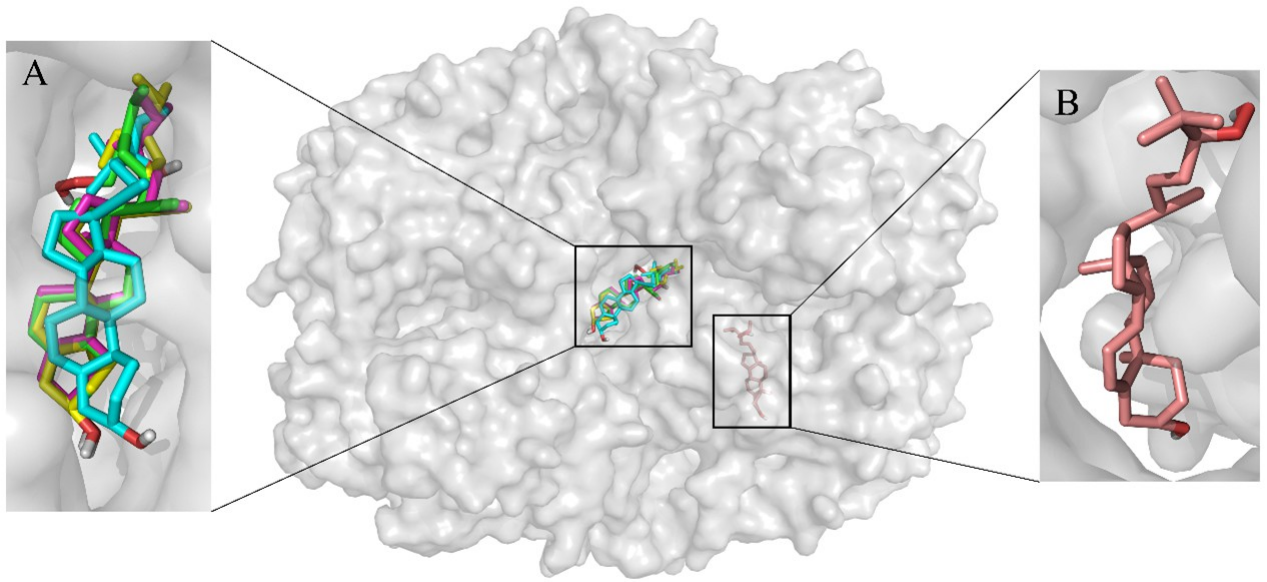
Molecular docking of COX-2 with compounds 1–5. Different compounds were located in different active pockets. Compounds 1, 2, 3 and 4 were located in the hydrophobic pocket A of COX-2 (Comp. 1 shown in green, Comp.2 shown in cyan, Comp.3 shown in magenta, Comp.4 shown in yellow), while compound 5 was located in the pocket B (Comp.5 shown in salmon).

**Fig 6:**
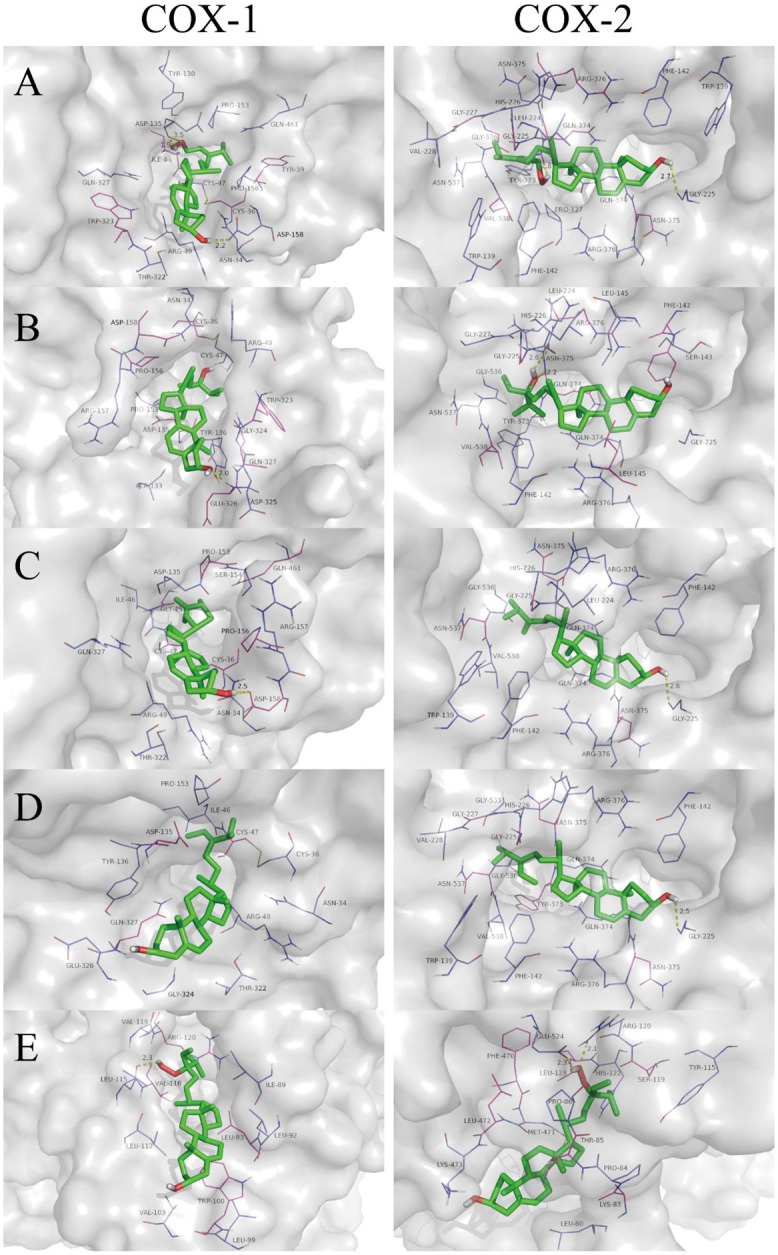
3-D docked poses of COX-1 and -2 with five sterols (compounds 1–5). The first column represents COX-1 and the second column represents COX-2. The rows present: A) compound 1, B) compound 2, C) compound 3, D) compound 4, E) compound 5.

The possible mechanisms beneath the inhibition of compounds 1–5 against COX-1 and -2 was presented through molecular docking (Figs 4–6, Table 1). As shown in Fig 4, compounds 1–5 were predicted to be located in different pockets of COX-1 (pocket A, B and C), as reported in previous studies [34]. The hydrogen bonds and interaction residues were displayed in 3D docked poses of compounds 1–5 binding to COX-1 and COX-2 (Fig 6). Compounds 1 and 4 located in the same active pocket of COX-1, which was surrounded by active site at ILE-46, CYS-47, CYS-36, ASN-34, ARG-49, THR-322, GLY-324, GLU-326, GLN-327, TYR-136, ASP-135 and PRO-153. The docking results also revealed that compound 1 typically bind within the COX-1 channel via H-bonding interactions with the side chain of ASP-135 and ASP-158, while compound 4 was embedded in the active pocket of COX-1 primarily through hydrophobic interactions (Fig 6, Table 1).

Furthermore, the binding sites and interactions between compounds 1–5 and COX-2 are represented in Figs 5 and 6. Compounds 1–4 were located in the hydrophobic pocket A of COX-2 (surrounded by active site at GLY-225, ASN-375, ARG-376, PHE-142, VAL-538, ASN-537, GLY-227, GLY-536, LEU-224, HIS-226, GLN-374, TYR-373), while compound 5 was located in the pocket B (surrounded by active site at PRO-84, LYS-83, LEU-80, LYS-473, LEU-472, PHE-470, GLU-524, ARG-120, TYR-115, THR-85, MET-471, PRO-86, LEU-123, HIS-122, SER-119). All of the five compounds had interactions with surrounding residues in the active site of COX-2. Compounds 1, 3 and 4 showed possible hydrogen bond with GLY-225, predicting which might be favorable binding site of COX-2 for sterols (Fig 6). Differ from compounds 1–4, compound 5 bound with COX-2 at another active pocket and showed possible hydrogen bonds with ARG-120 and GLU-524. Moreover, there was a high binding energy between compound 4 and COX-2 (-9.02 kcal/mol), which was comparable to that of the compound 5 (-6.97 kcal/mol).

COX-1 and -2 are bifunctional enzymes that convert arachidonic acid (AA) to prostaglandin G_2_ (PGG_2_) in their cyclooxygenase active site [35]. Our results suggested that fucosterol (compound 4) was located in the hydrophobic pocket of COX-1 and -2 enzyme with the highest binding energy (-7.85 and -9.02 kcal/mol) in molecular docking, compared to the other sterols. The possible mechanism underlying the highest COX-1 and -2 enzyme inhibitory activities of fucosterol might be attributed to the distinct olefin methine presented in its molecular structure, which could occupy the active site of the enzyme and prevent the enzyme from contacting with the substrate. This is in agreement with previous molecular docking results that tetracyclic skeletons and incorporation of an aliphatic chain in sterols were the key structural requirements theoretically for their good COX-1 and COX-2 binding activity [36]. Thus, these findings confirmed that fucosterol with tetracyclic skeletons and olefin methine achieved the good COX-1 and -2 enzyme inhibitory activities through hydrophobic interactions and hydrogen bond.

## 4. Conclusions

Purification of hexane extracts from *L*. *japonica* afforded 5 sterols, and their structures were identified using spectroscopic and chemical evidence. Spectroscopic methods characterized these compounds as 29-Hydroperoxy-stigmasta-5,24(28)-dien-3β-ol (compound 1), saringosterol (24-vinyl-cholest-5-ene-3β,24-diol) (compound 2), 24-methylenecholesterol (compound 3), fucosterol (stigmasta-5,24-diene-3β-ol) (compound 4), and 24-Hydroperoxy-24-vinyl-cholesterol (compound 5). These pure isolates were tested for antioxidant and anti-inflammatory activities using LPO and COX-1 and -2 enzyme inhibitory assays. Compared to the other compounds, fucosterol (compound 4) exhibited the highest COX-1 and -2 enzyme inhibitory activities at 59 and 47%, respectively. The COX enzyme inhibitory activity of 24-methylenecholesterol (compound 3) and fucosterol (compound 4) was comparable to the activity of the NSAIDs aspirin and ibuprofen. For the LPO assays, saringosterol (compound 2), 24-methylenecholesterol (compound 3) and fucosterol (compound 4) showed higher LPO inhibitory activity at >50% than the other compounds. In addition, the results of molecular docking predicted that 5 sterols were located in different pocket of COX-1 and -2 and confirmed that fucosterol with tetracyclic skeletons and olefin methine achieved the best COX-1 and -2 enzyme inhibitory activities through hydrophobic interactions and hydrogen bond. Our results confirm the presence of 5 sterols in *L*. *japonica* and its significant anti-inflammatory and antioxidant activity. Therefore, it appears that *L*. *japonica* have the potential to be developed as a dietary supplement, but molecular tests are required to verify the activity of 5 sterols in further study.

## Supporting information

S1 Fig HPLC profile of fraction LJ-1. (DOCX)

S2 Fig HR-ESITOFMS (positive) of 1. (DOCX)

S3 Fig ^1^H NMR spectrum of 1 in CDCl_3_. (DOCX)

S4 Fig ^13^C NMR spectrum of 1 in CDCl_3_. (DOCX)

S5 Fig HR-ESITOFMS (positive) of 2. (DOCX)

S6 Fig ^1^H NMR spectrum of 2 in CDCl_3_. (DOCX)

S7 Fig ^13^C NMR spectrum of 2 in CDCl_3_. (DOCX)

S8 Fig HR-ESITOFMS (positive) of 3. (DOCX)

S9 Fig ^1^H NMR spectrum of 3 in CDCl_3_. (DOCX)

S10 Fig ^13^C NMR spectrum of 3 in CDCl_3_. (DOCX)

S11 Fig HR-ESITOFMS (positive) of 4. (DOCX)

S12 Fig ^1^H NMR spectrum of 4 in CDCl_3_. (DOCX)

S13 Fig ^13^C NMR spectrum of 4 in CDCl_3_. (DOCX)

S14 Fig HR-ESITOFMS (positive) of 5. (DOCX)

S15 Fig ^1^H NMR spectrum of 5 in CDCl_3_. (DOCX)

S16 Fig ^13^C NMR spectrum of 5 in CDCl_3_. (DOCX)

S17 Fig Lipid peroxidation (LPO) inhibitory activities of sterols (1–5) isolated from the hexane extract of *L*. *japonica* and commercial cholesterol tested at 25 μg/mL. (DOCX)

S18 Fig Overlay of five sterols’ (compounds 1–5) conformations (shown in green) with their best docked conformations (shown in cyan) in COX-1 and -2. (DOCX)

S1 Graphical abstract (TIF)

## Abbreviations

AA: arachidonic acid
BHA: butylated hydroxyanisole
BHT: butylated hydroxytoluene
COX: cyclooxygenase
HPLC: high pressure liquid chromatography
HRESITOFMS: high-resolution electrospray ionization mass spectrometry
LPO: lipid peroxidation
LUVs: large unilamellar vesicles
MPLC: medium-pressure liquid chromatography
NMR: nuclear magnetic resonance
NSAIDs: nonsteroidal anti-inflammatory drugs
Prep-TLC: preparative thin-layer chromatography
SLPC: 1-stearoyl-2-linoleoyl-sn-glycerol-3-phosphocholine
TBHQ: t-butylhydroquinone
VLC: vacuum liquid chromatography
