# Supplementary material for: The edible seaweed Laminaria japonica contains cholesterol analogues that inhibit lipid peroxidation and cyclooxygenase enzymes
Source: PLoS One. 2022 Jan 27;17(1):e0258980. doi: 10.1371/journal.pone.0258980 (PMC8794173; doi:10.1371/journal.pone.0258980)
Supplement: S1 Fig — (DOCX) [file pone.0258980.s001.docx]

**
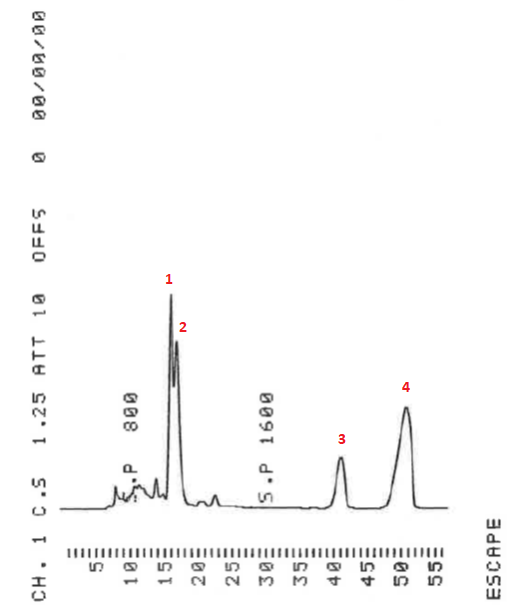
**

**S1 Fig**. HPLC profiles of the fraction LJ-1. Solvent system: acetonitrile: methanol, 50:50 at 26 °C on a C-18 preparative HPLC column (Xtera, Waters Corp.). Flow rate 4.0 mL/min and peaks monitored at 210 nm. **1** (29-Hydroperoxy-stigmasta-5,24(28)-dien-3β-ol), **2** (24-vinyl-cholest-5-ene-3β,24-diol), **3** (24-methylenecholesterol) and **4** (fucosterol, stigmasta-5,24-diene-3β-ol).
