# Supplementary figures and images for: The edible seaweed Laminaria japonica contains cholesterol analogues that inhibit lipid peroxidation and cyclooxygenase enzymes
Source: PLoS One. 2022 Jan 27;17(1):e0258980. doi: 10.1371/journal.pone.0258980 (PMC8794173; doi:10.1371/journal.pone.0258980)

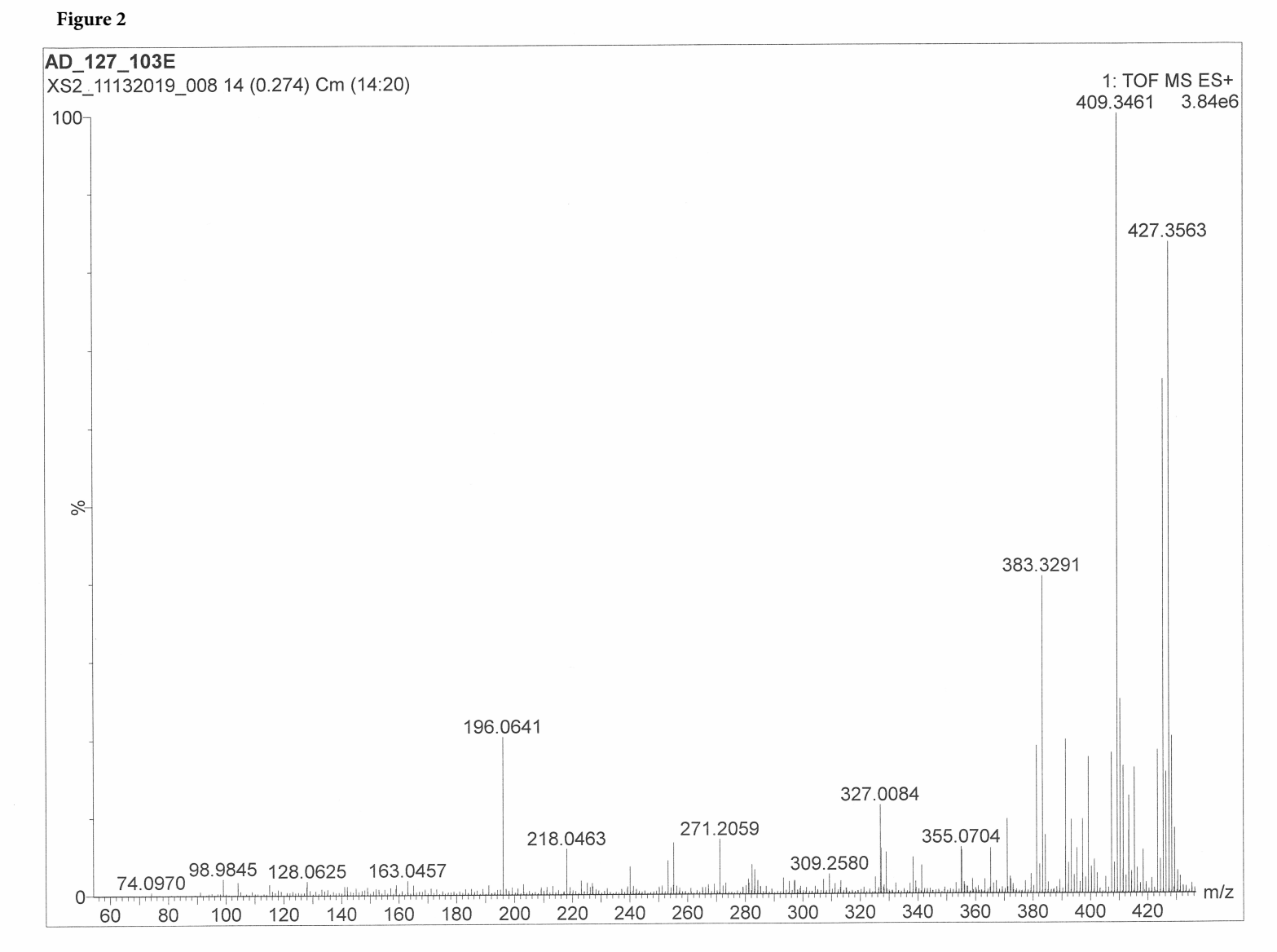


**S2 Fig**. HR-ESITOFMS (positive) of **1**

Supplement: S2 Fig — (DOCX) [file pone.0258980.s002.docx]

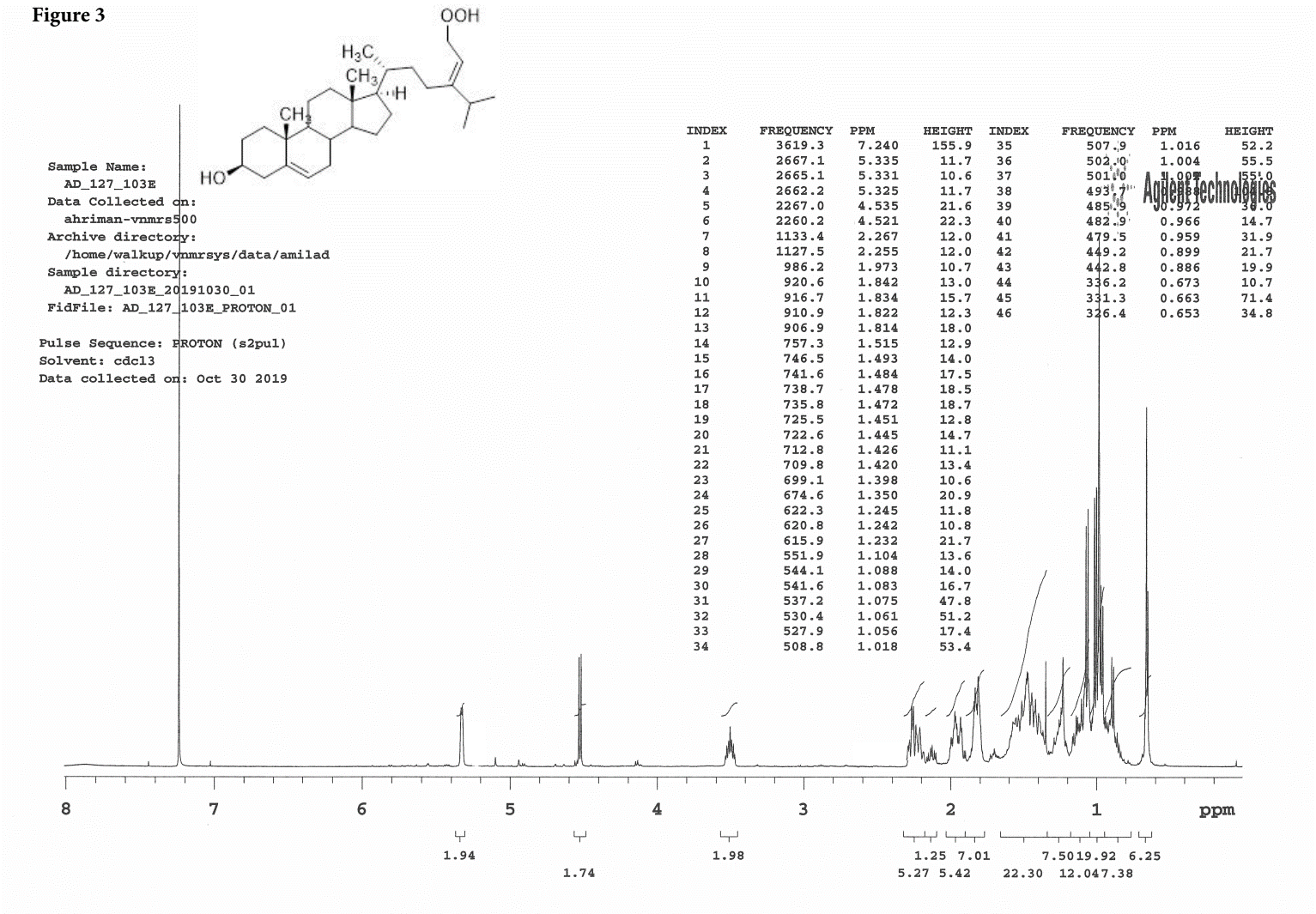


**S3 Fig**. 1H NMR spectrum of **1** in CDCl3

Supplement: S3 Fig — (DOCX) [file pone.0258980.s003.docx]

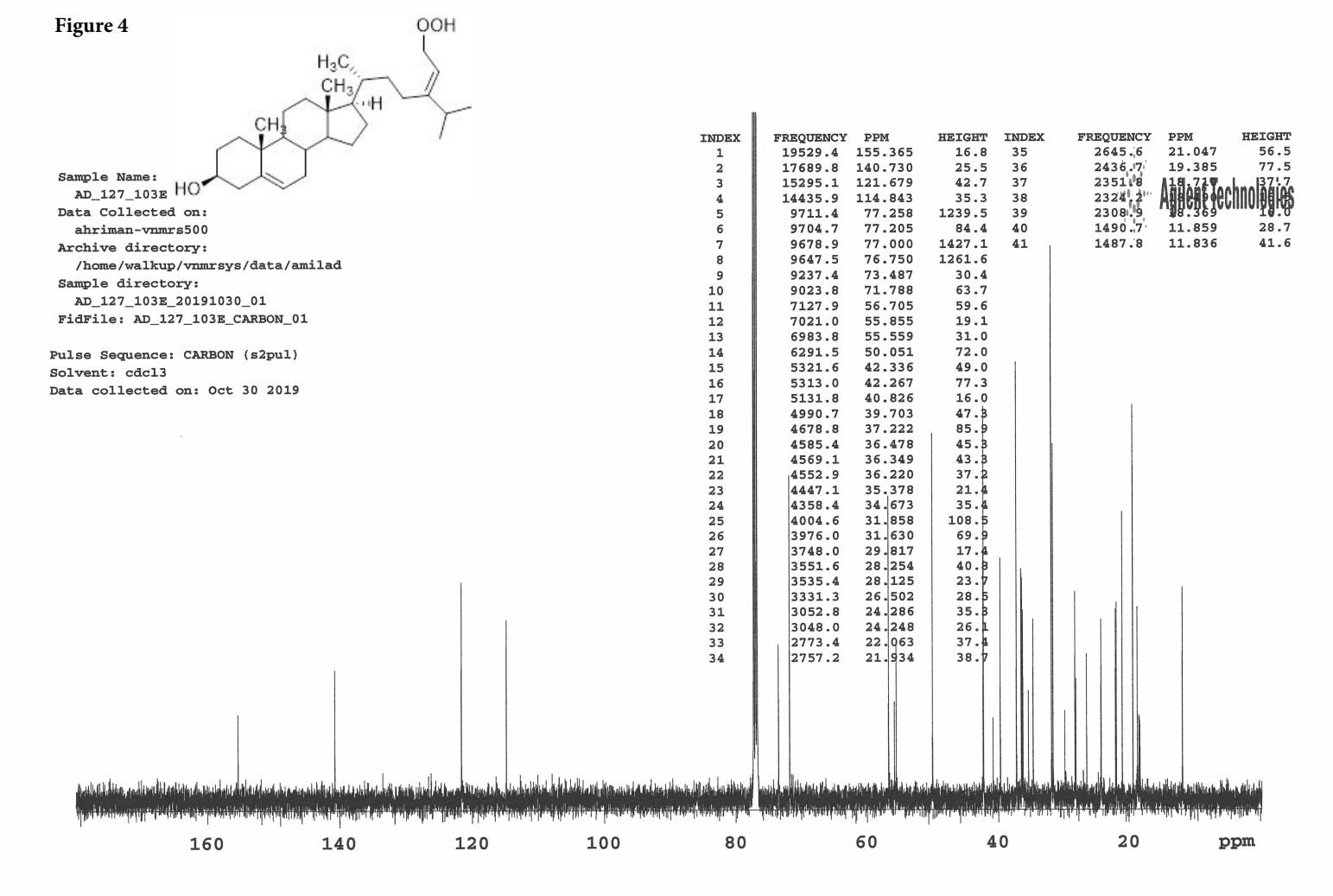


**S4 Fig**. 13C NMR spectrum of **1** in CDCl3

Supplement: S4 Fig — (DOCX) [file pone.0258980.s004.docx]

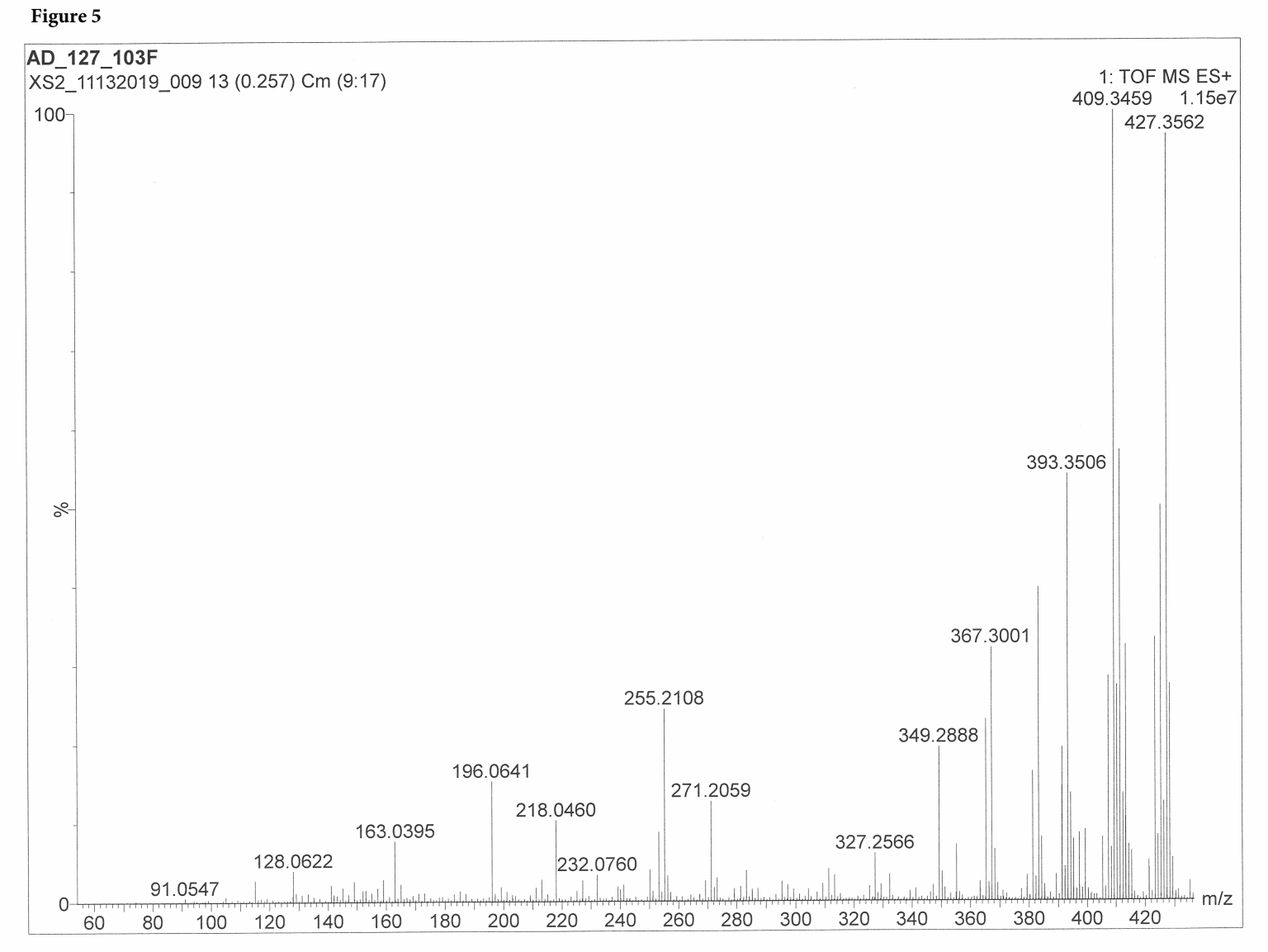


**S5 Fig**. HR-ESITOFMS (positive) of **2**

Supplement: S5 Fig — (DOCX) [file pone.0258980.s005.docx]

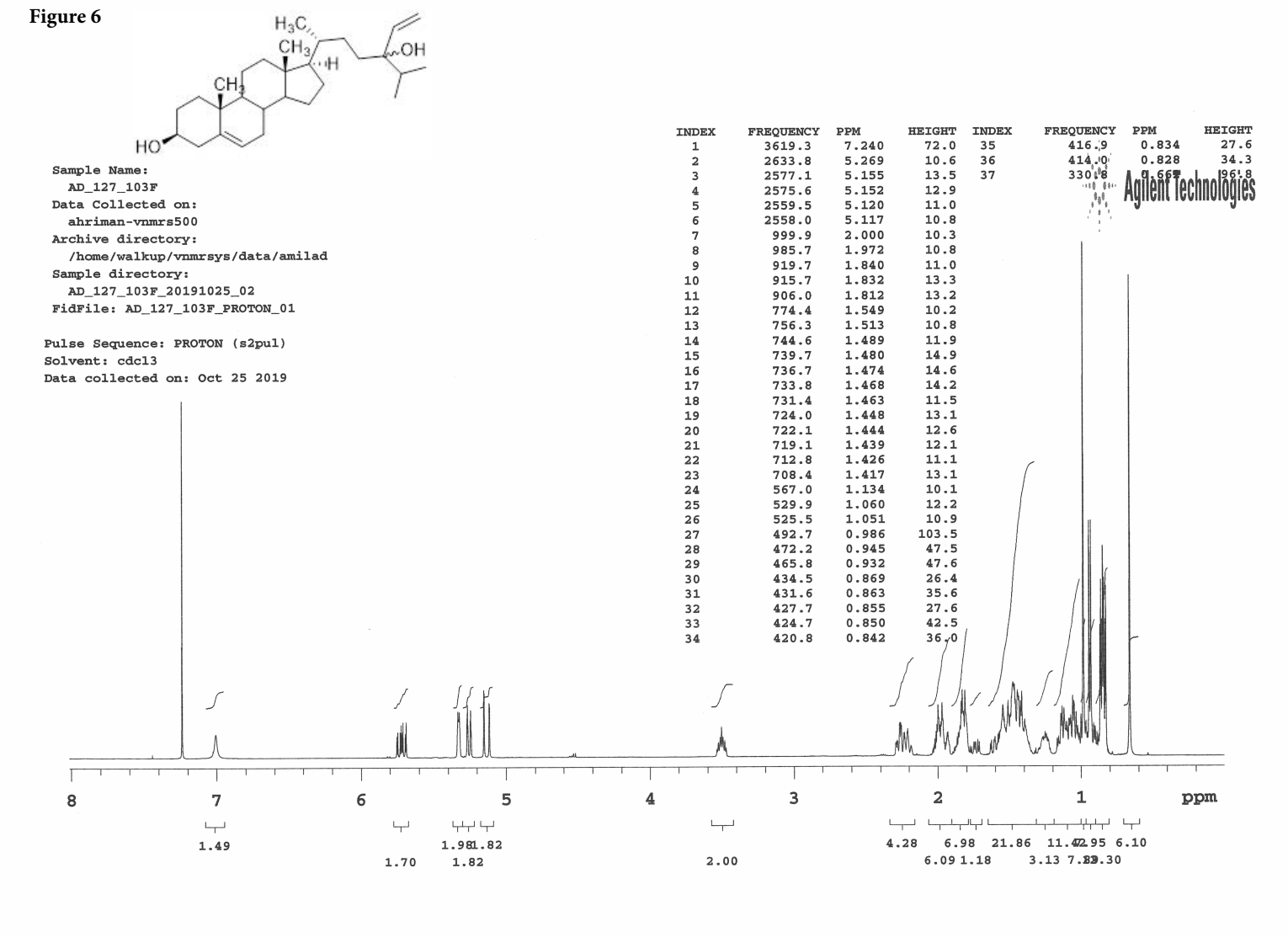


**S6 Fig**. 1H NMR spectrum of **2** in CDCl3

Supplement: S6 Fig — (DOCX) [file pone.0258980.s006.docx]

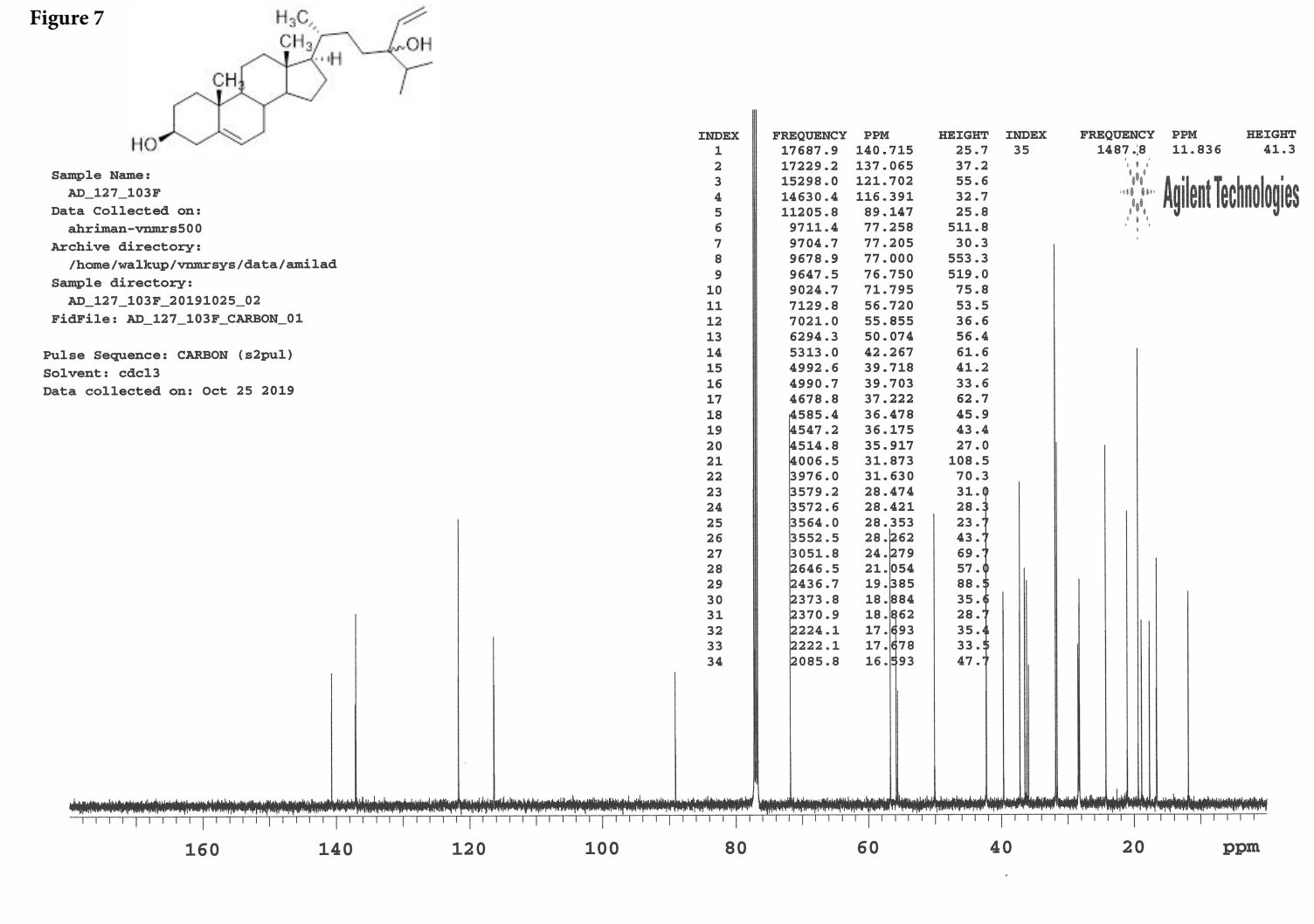


**S7 Fig**. 13C NMR spectrum of **2** in CDCl3

Supplement: S7 Fig — (DOCX) [file pone.0258980.s007.docx]

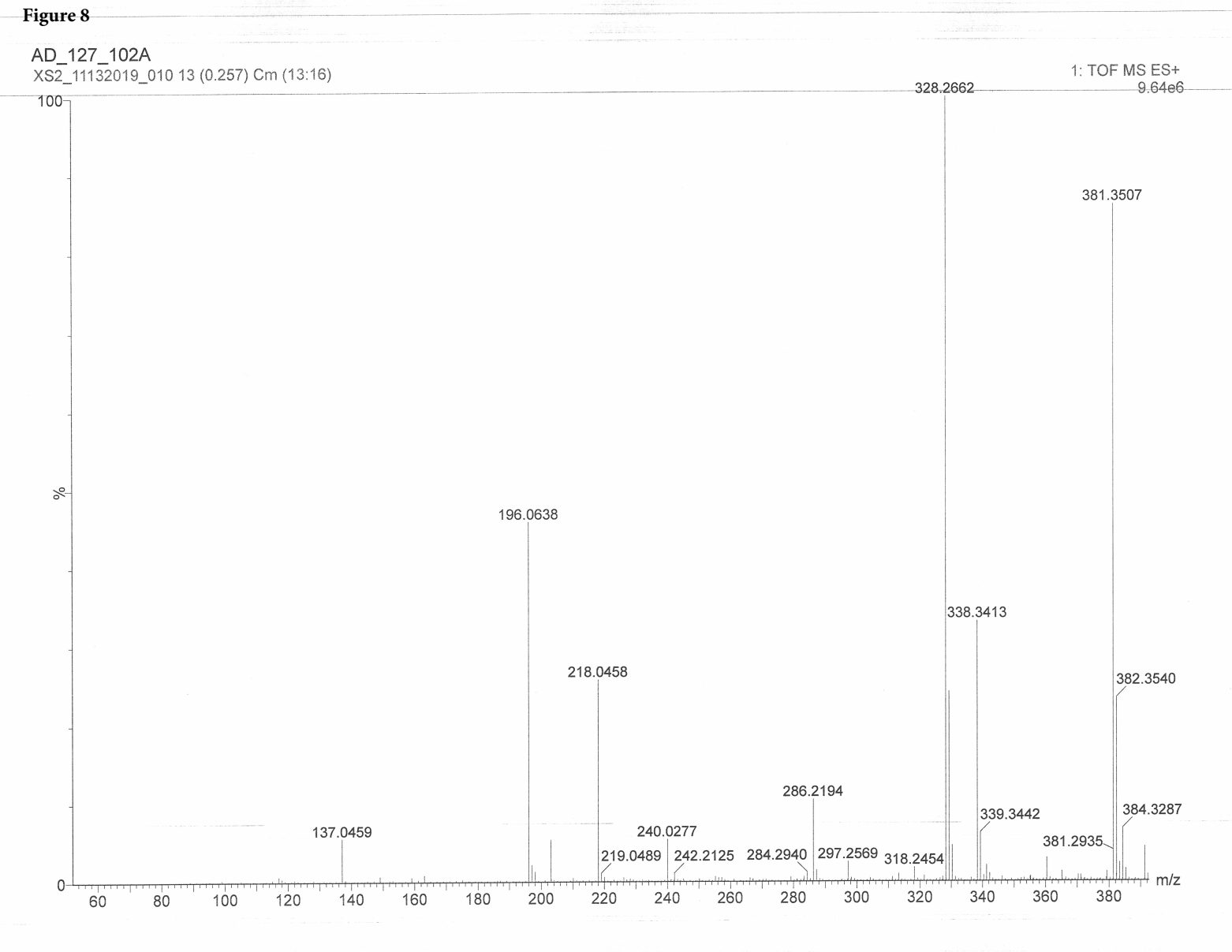


**S8 Fig**. HR-ESITOFMS (positive) of **3**

Supplement: S8 Fig — (DOCX) [file pone.0258980.s008.docx]

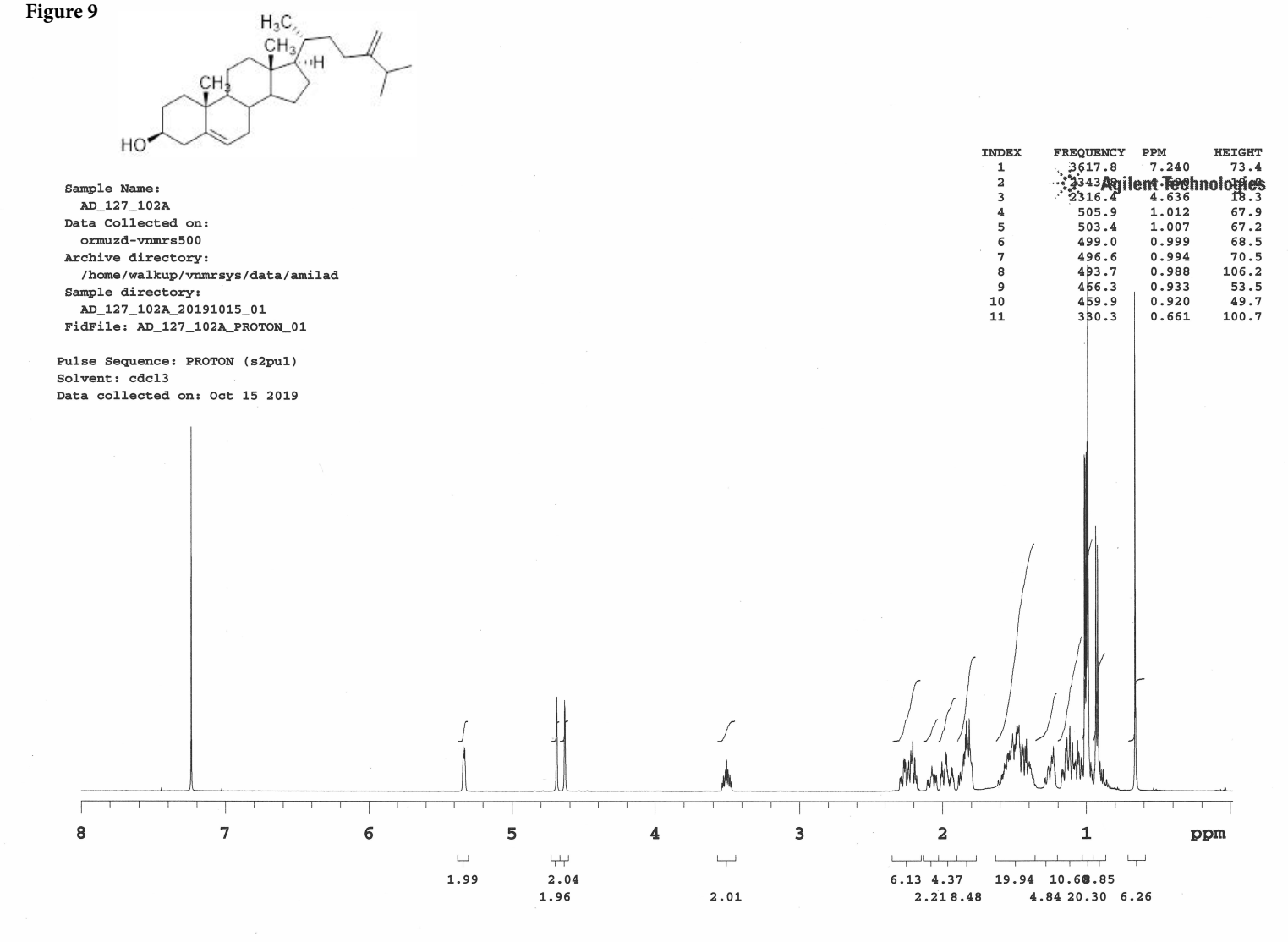


**S9 Fig**. 1H NMR spectrum of **3** in CDCl3

Supplement: S9 Fig — (DOCX) [file pone.0258980.s009.docx]

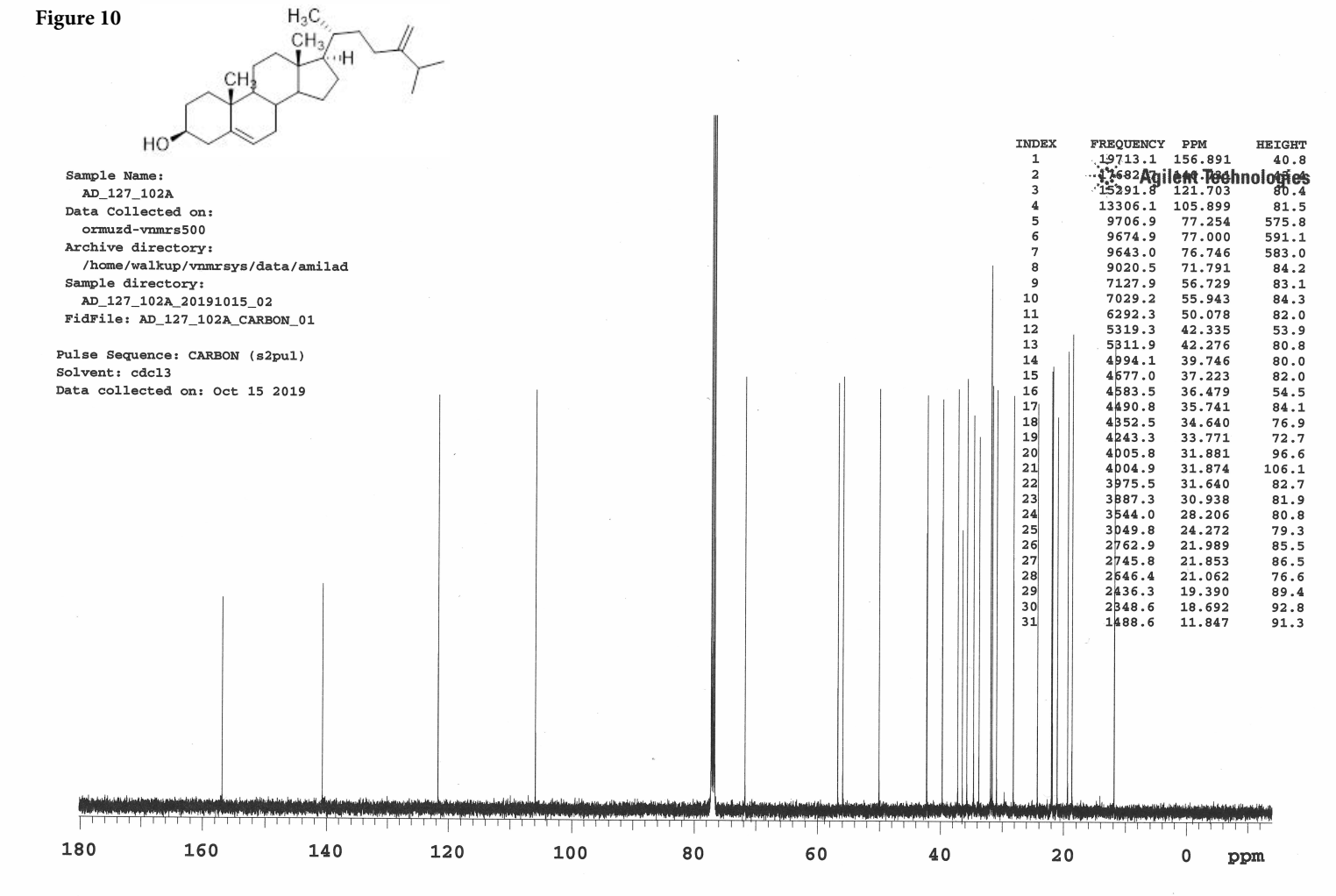


**S10 Fig**. 13C NMR spectrum of **3** in CDCl3

Supplement: S10 Fig — (DOCX) [file pone.0258980.s010.docx]

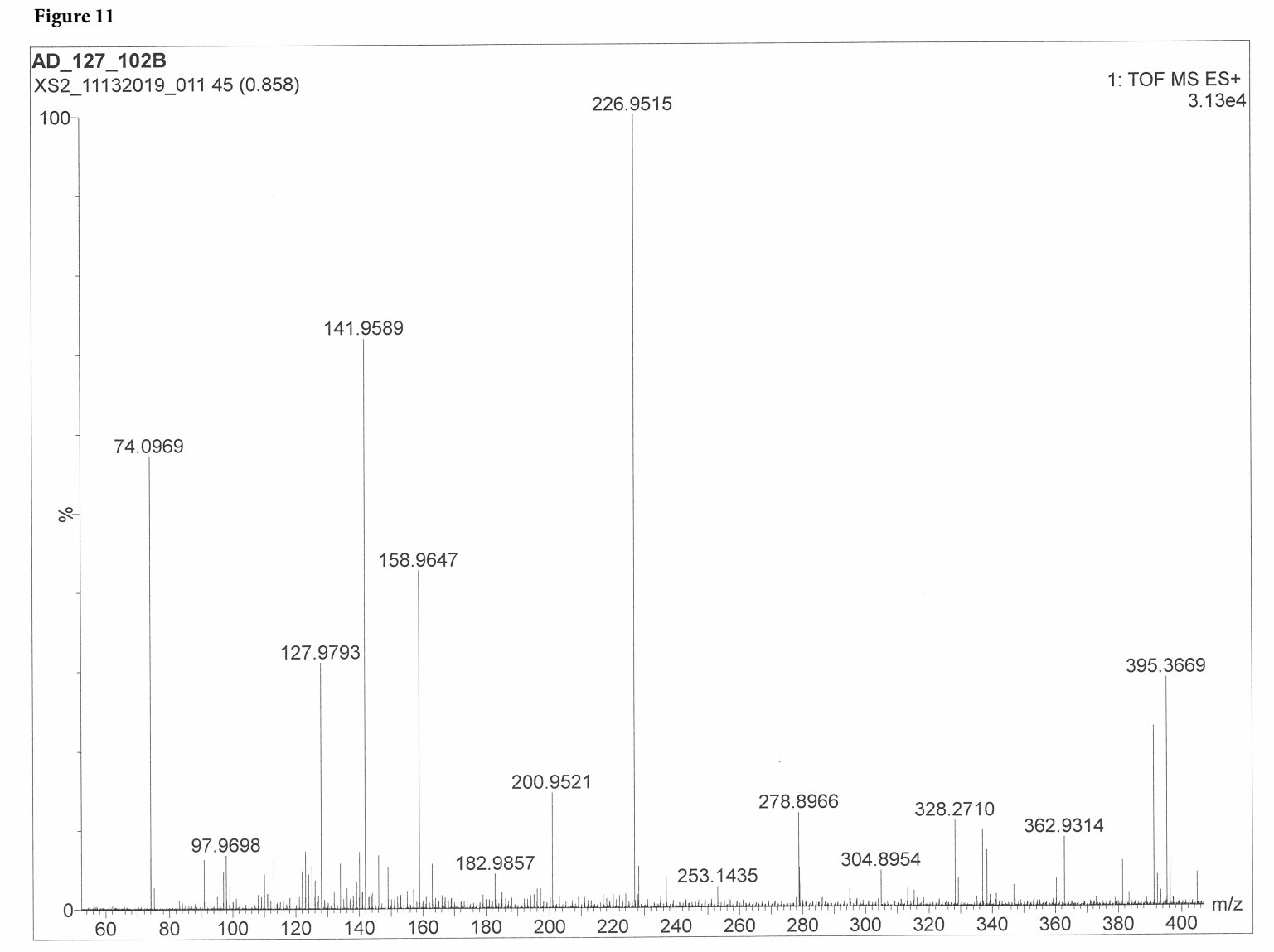


**S11 Fig**. HR-ESITOFMS (positive) of **4**

Supplement: S11 Fig — (DOCX) [file pone.0258980.s011.docx]

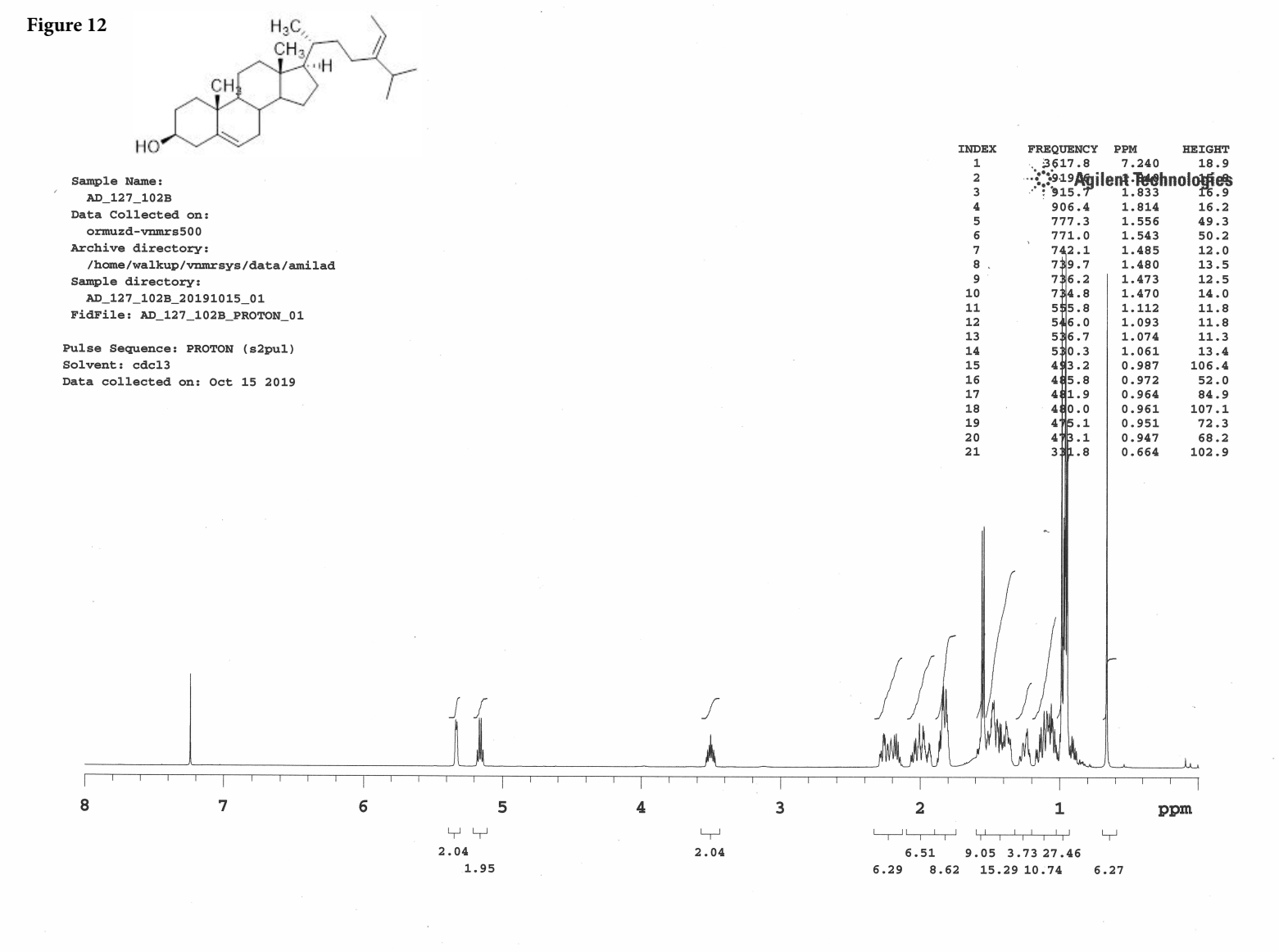


**S12 Fig**. 1H NMR spectrum of **4** in CDCl3

Supplement: S12 Fig — (DOCX) [file pone.0258980.s012.docx]

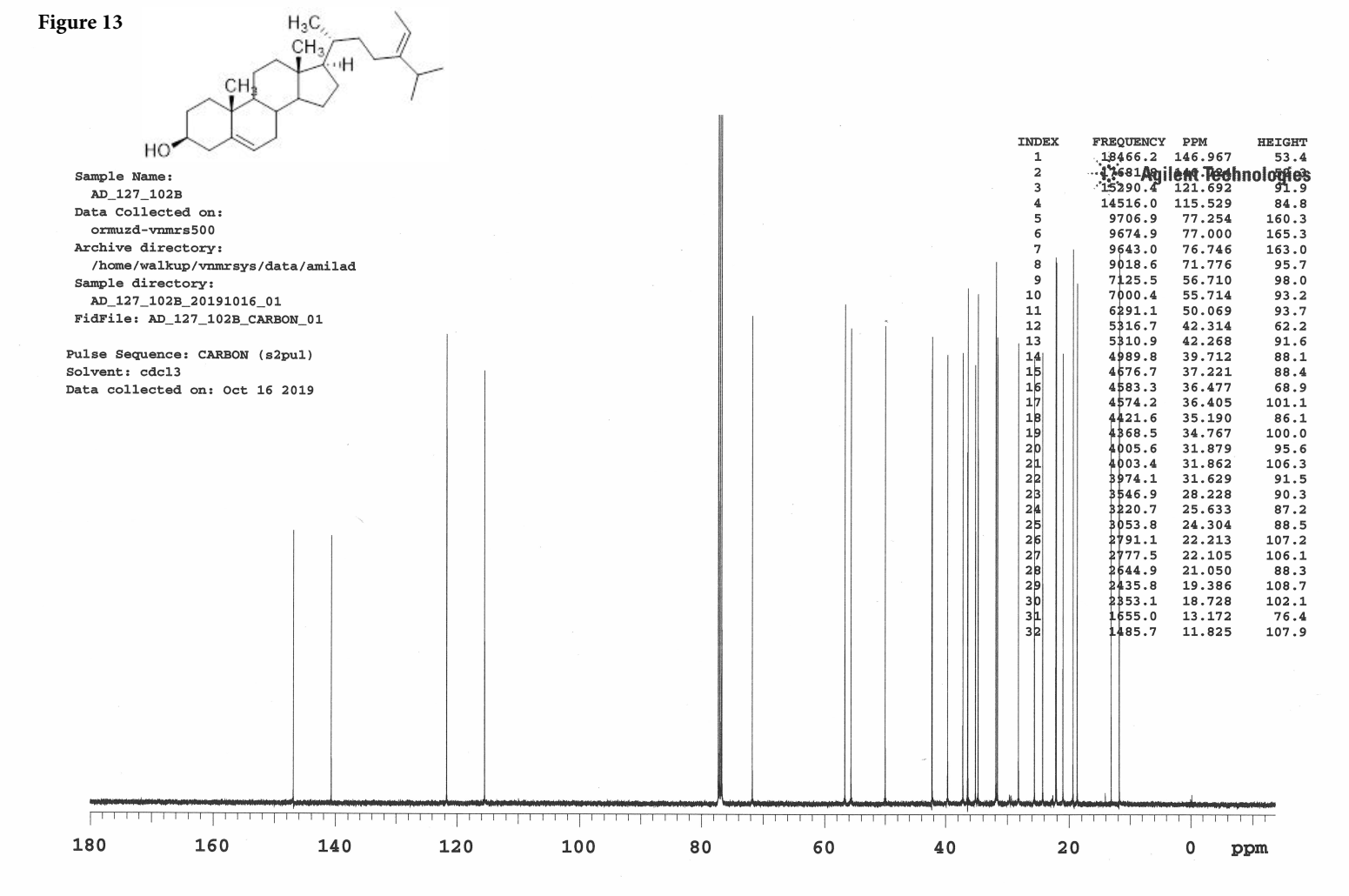


**S13 Fig**. 13C NMR spectrum of **4** in CDCl3

Supplement: S13 Fig — (DOCX) [file pone.0258980.s013.docx]

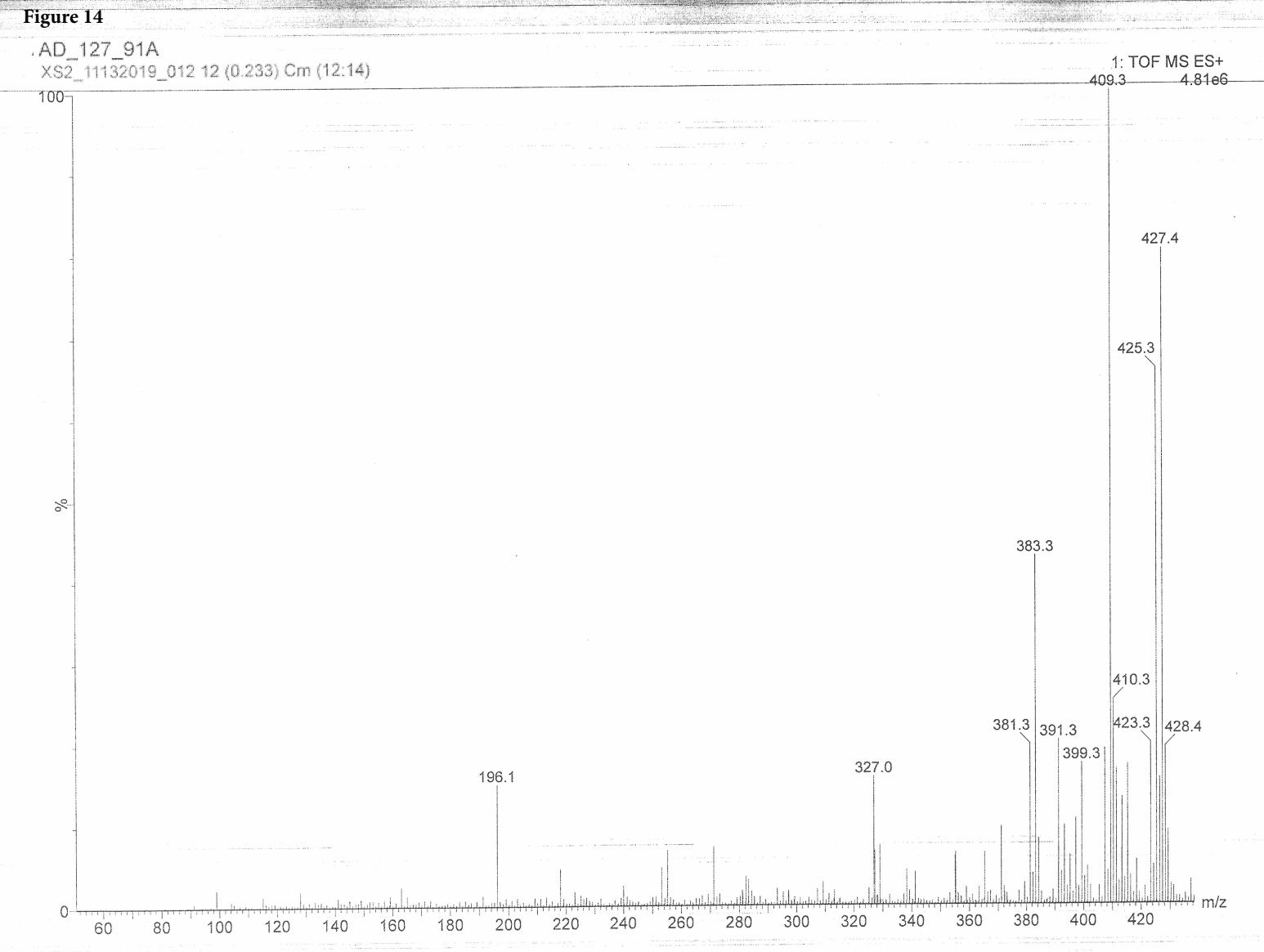


**S14 Fig**. HR-ESITOFMS (positive) of **5**

Supplement: S14 Fig — (DOCX) [file pone.0258980.s014.docx]

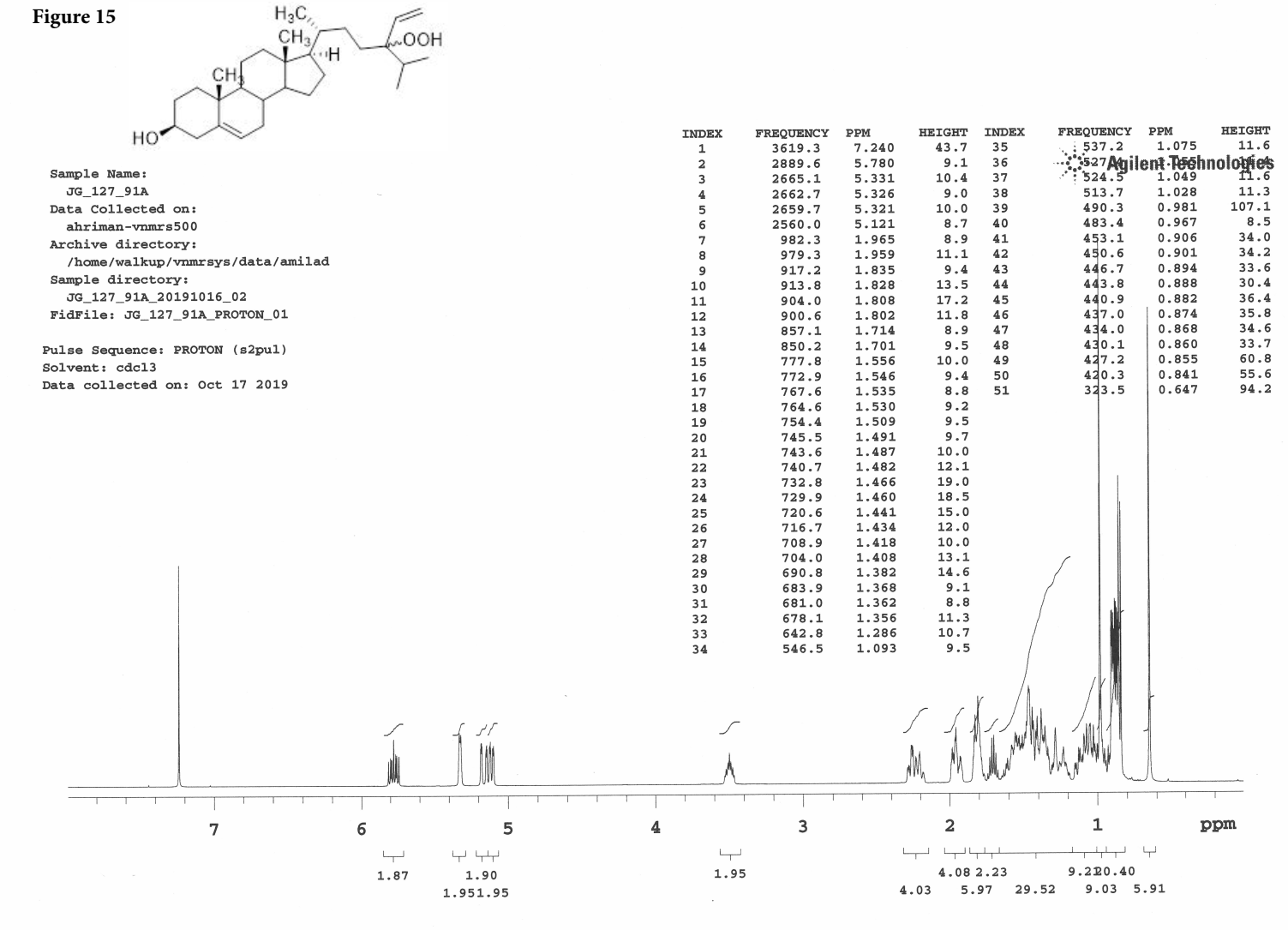


**S15 Fig**. 1H NMR spectrum of **5** in CDCl3

Supplement: S15 Fig — (DOCX) [file pone.0258980.s015.docx]

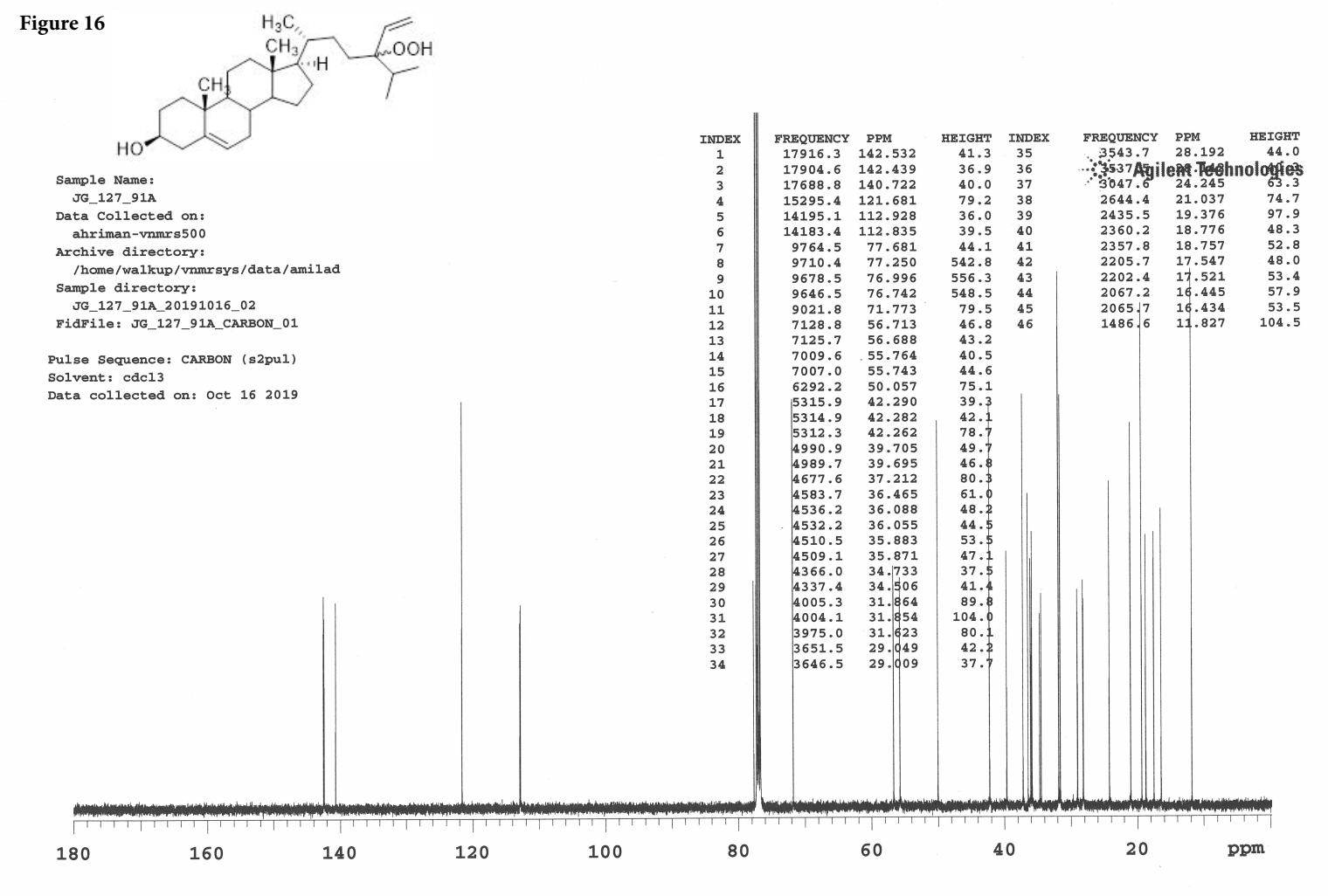


**S16 Fig**. 13C NMR spectrum of **5** in CDCl3

Supplement: S16 Fig — (DOCX) [file pone.0258980.s016.docx]

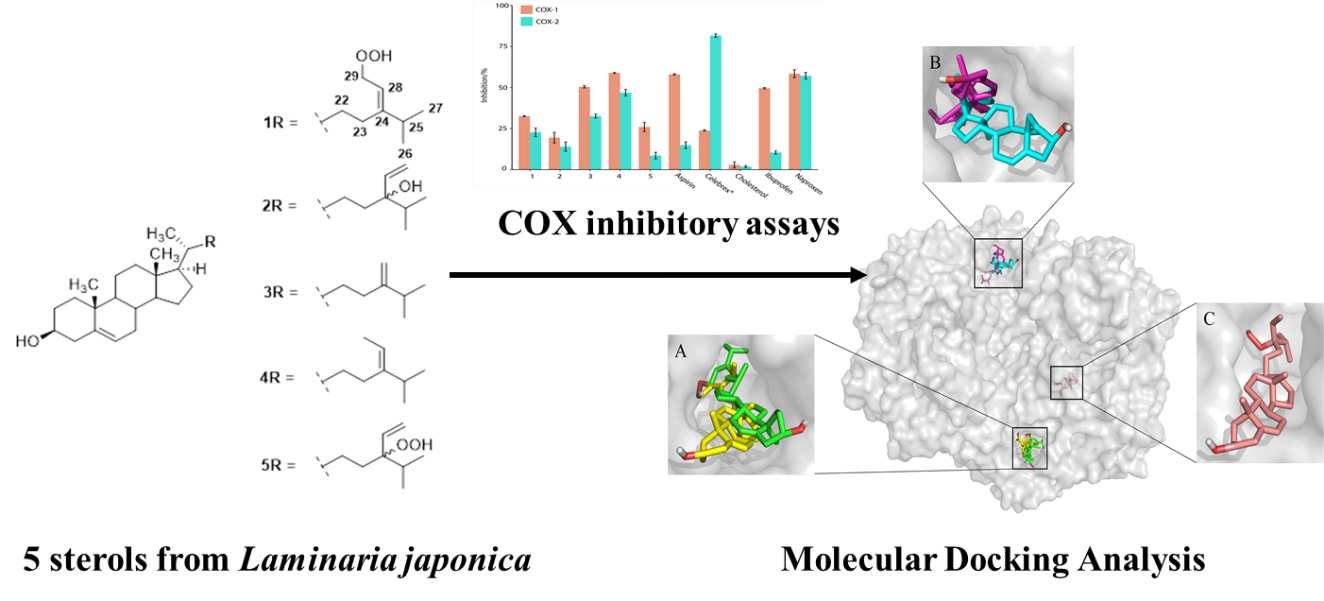

Supplement: S1 Graphical abstract — (TIF) [file pone.0258980.s019.tif]
