# Supplementary material for: The edible seaweed Laminaria japonica contains cholesterol analogues that inhibit lipid peroxidation and cyclooxygenase enzymes
Source: PLoS One. 2022 Jan 27;17(1):e0258980. doi: 10.1371/journal.pone.0258980 (PMC8794173; doi:10.1371/journal.pone.0258980)
Supplement: S17 Fig — (DOCX) [file pone.0258980.s017.docx]

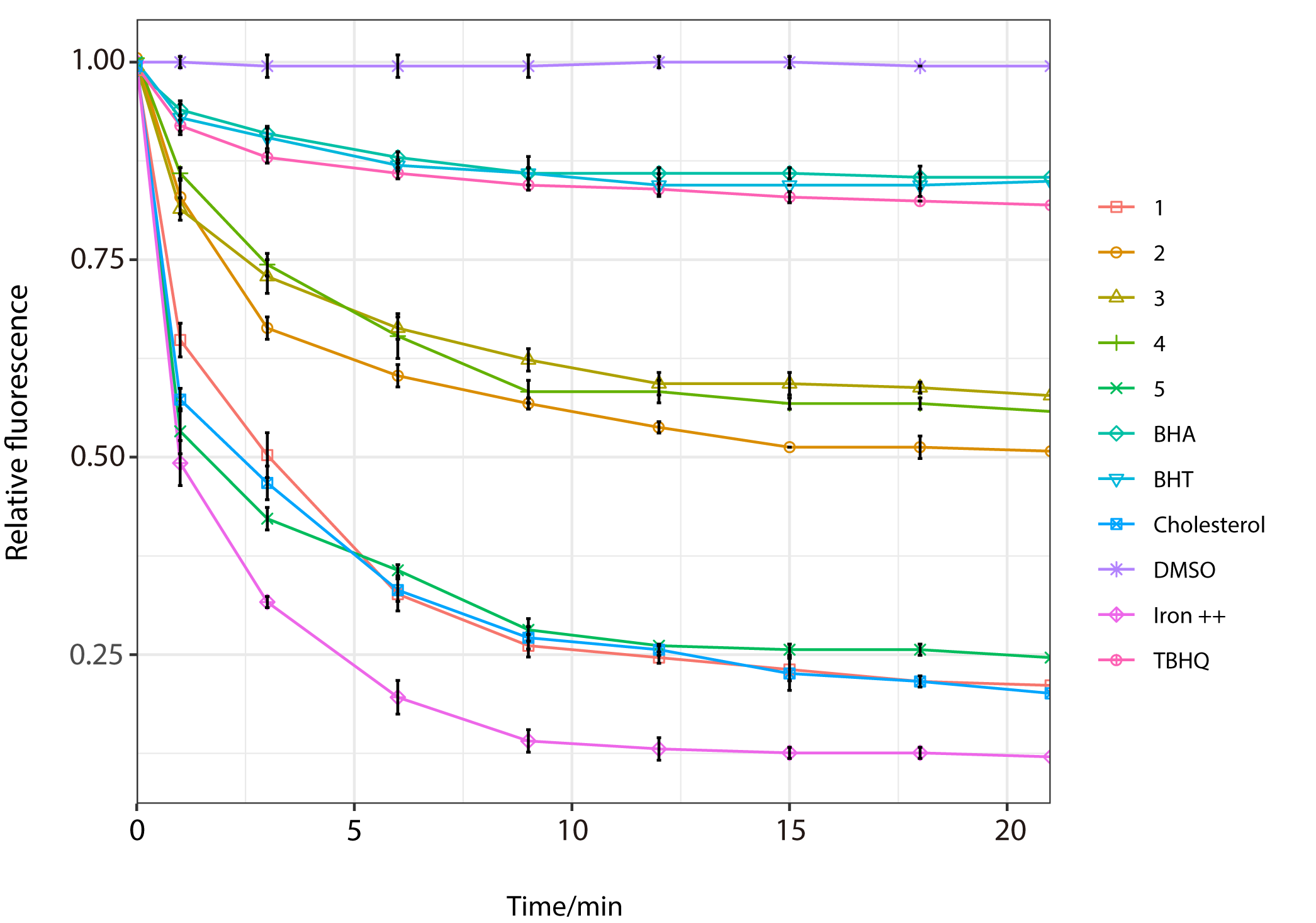


**S17 Fig**. Lipid peroxidation (LPO) inhibitory activities of sterols (1-5) isolated from the hexane extract of L. japonica and commercial cholesterol tested at 25 μg/mL. Commercial antioxidants BHA, BHT and TBHQ used as positive controls at 1.802, 2.204 and 1.662 µg/mL. The oxidation of lipid was initiated by the addition of Fe2+ ions. The varying concentrations of positive controls used were to yield a comparable activity profiles between 50-100% by test compounds and positive controls alike. Vertical bars represent the standard deviation of each data point (n=2).
