# Supplementary material for: The edible seaweed Laminaria japonica contains cholesterol analogues that inhibit lipid peroxidation and cyclooxygenase enzymes
Source: PLoS One. 2022 Jan 27;17(1):e0258980. doi: 10.1371/journal.pone.0258980 (PMC8794173; doi:10.1371/journal.pone.0258980)
Supplement: S18 Fig — (DOCX) [file pone.0258980.s018.docx]

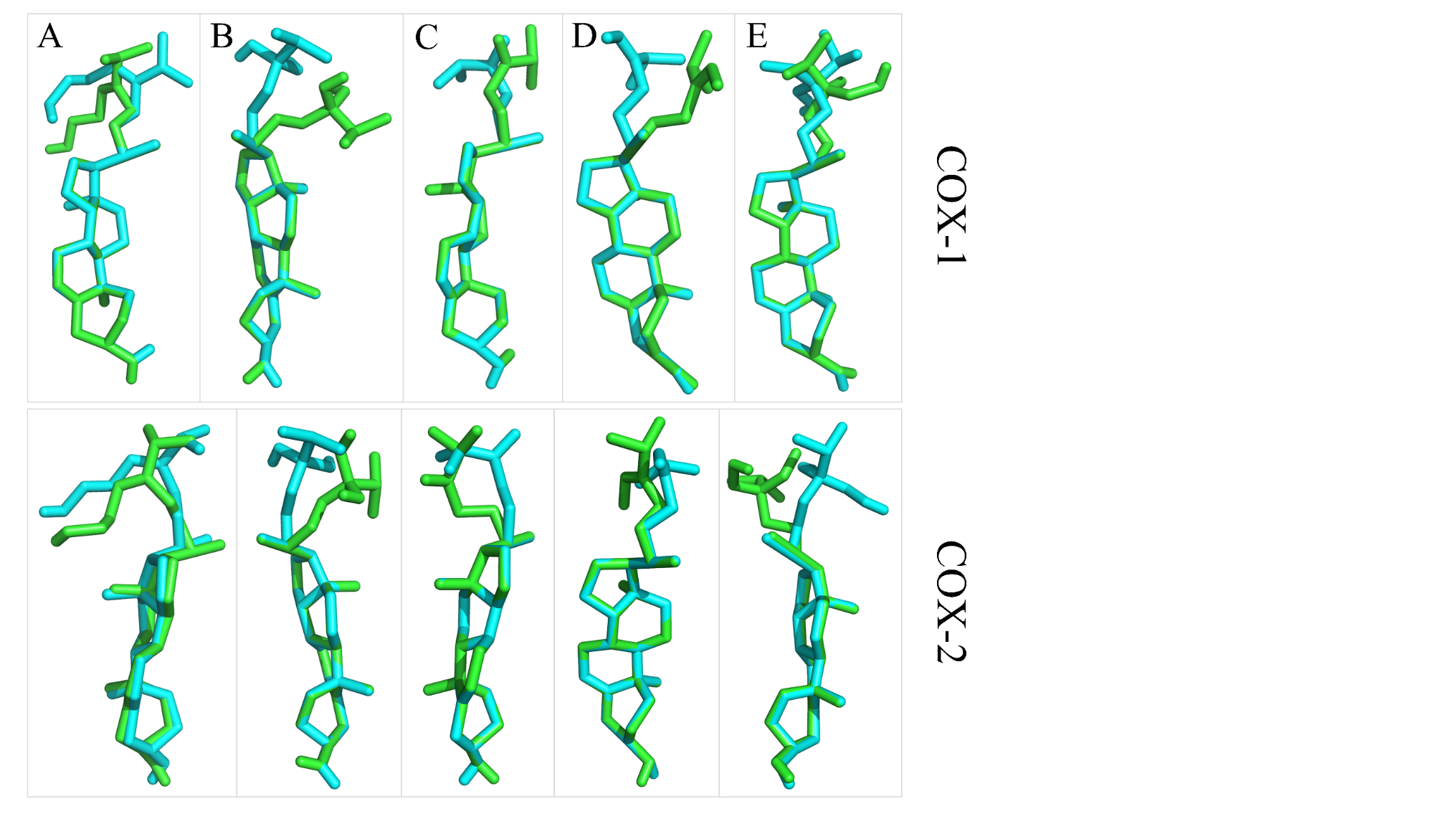


**S18 Fig**. Overlay of five sterols’ (compounds 1-5) conformations (shown in green) with their best docked conformations (shown in cyan) in COX-1 and -2. The first row represents COX-1 and the second row represents COX-2. The column present: A) compound 1, B) compound 2, C) compound 3, D) compound 4, E) compound 5.
